# Large-Scale Habitat Corridors for Biodiversity Conservation: A Forest Corridor in Madagascar

**DOI:** 10.1371/journal.pone.0132126

**Published:** 2015-07-22

**Authors:** Tanjona Ramiadantsoa, Otso Ovaskainen, Joel Rybicki, Ilkka Hanski

**Affiliations:** 1 Metapopulation Research Centre, Department of Biosciences, University of Helsinki, Helsinki, Finland; 2 Department of Computer Science, Aalto University, Espoo, Finland; 3 Department of Algorithms and Complexity, Max Planck Institute for Informatics, Saarbrücken, Germany; Northern Illinois University, UNITED STATES

## Abstract

In biodiversity conservation, habitat corridors are assumed to increase landscape-level connectivity and to enhance the viability of otherwise isolated populations. While the role of corridors is supported by empirical evidence, studies have typically been conducted at small spatial scales. Here, we assess the quality and the functionality of a large 95-km long forest corridor connecting two large national parks (416 and 311 km^2^) in the southeastern escarpment of Madagascar. We analyze the occurrence of 300 species in 5 taxonomic groups in the parks and in the corridor, and combine high-resolution forest cover data with a simulation model to examine various scenarios of corridor destruction. At present, the corridor contains essentially the same communities as the national parks, reflecting its breadth which on average matches that of the parks. In the simulation model, we consider three types of dispersers: passive dispersers, which settle randomly around the source population; active dispersers, which settle only in favorable habitat; and gap-avoiding active dispersers, which avoid dispersing across non-habitat. Our results suggest that long-distance passive dispersers are most sensitive to ongoing degradation of the corridor, because increasing numbers of propagules are lost outside the forest habitat. For a wide range of dispersal parameters, the national parks are large enough to sustain stable populations until the corridor becomes severely broken, which will happen around 2065 if the current rate of forest loss continues. A significant decrease in gene flow along the corridor is expected after 2040, and this will exacerbate the adverse consequences of isolation. Our results demonstrate that simulation studies assessing the role of habitat corridors should pay close attention to the mode of dispersal and the effects of regional stochasticity.

## Introduction

Habitat loss and fragmentation is the principal cause of biodiversity decline locally and globally [1]. Two well-justified measures to protect biodiversity are to increase the amount of protected areas and to ameliorate conditions in the existing protected areas [2–4]. The latter involves possible habitat restoration but also the establishment of corridors to enhance the conservation value of the protected areas (Aichi Biodiversity Target 11). Backed by theoretical foundations in the theory of island biogeography [5] and in metapopulation theory [6], which emphasize the role of landscape structure for the persistence of species, corridors are expected to increase connectivity between protected areas and other habitat fragments, and thereby to reduce local extinction rate, to facilitate re-colonization of currently unoccupied areas, as well as to reduce adverse genetic effects of isolation. Corridors have been defined in various ways, including movement corridors, habitat corridors and linear or stepping-stone corridors, but the ultimate aim in all cases is to enhance the viability of populations and communities which they connect.

It is easy to see the value of corridors in theory, but how effective they are in practice remains a contentious issue in part because direct evidence remains scant [7–10]. The factor that has been addressed in many studies is the role of corridors in facilitating and directing movements [11–15]. However, studies focused only on movements do not suffice to demonstrate the expected positive effect of habitat corridors on long-term viability of populations [8]. To do that, numerous experimental studies have been conducted (e.g. [16–18]), but a limitation of these studies is the typically very small spatial scale, from tens of centimeter up to some hundreds of meters, which makes it difficult to extrapolate conclusions to natural landscapes. Moreover, many of the small-scale experiments have not focused on species that would be of real interest in conservation. Reviews focusing on the effectiveness of corridors have emphasized that any results on the functionality of corridors are likely to be specific to particular study systems and should be applied only with caution to other systems [8, 19].

Observational studies have concluded that corridors have a positive effect on the occurrence of populations, consistent with numerous studies documenting positive effects of connectivity on the occurrence of species in networks of habitat fragments [6, 20]. For instance, in Uganda encroachment of the corridor connecting the Kibale forest and the Queen Elisabeth National Park has resulted in a decline in the size of the elephant population [21]. Dixon et al. [22] demonstrated the functionality of the Osceola-Ocala corridor in Florida in enhancing gene flow of the black bear population across the entire region. These studies involve large-scale habitat corridors that are tens to a hundred kilometers long. Large-scale corridors can be expected to be effective because not only do they facilitate movements but they also provide additional habitat. Very ambitious continent-scale corridor networks have been proposed, such as the Algonquin to Adirondack Initiative [23], Southern Rockies Ecosystem Project [24], Yellowstone to Yukon Conservation [25], and a large-scale forest corridor in Fennoscandia [26].

Madagascar is one of the biodiversity hotspots with high species richness and exceptionally high endemism (e.g. [27]), but Malagasy biota is also highly threatened. Extensive slash-and-burn agriculture and cattle grazing coupled with an ever increasing population has resulted in rapid deforestation [28]. Estimates of the original amount of forest prior to human arrival vary widely [29, 30], but recent estimates suggest that during the last 50 years alone forest cover has declined by almost 40% [31]. Deforestation has also increased forest fragmentation. For instance, large blocks of forest with area greater than 10 000 km^2^ comprised more than 60% of the forest cover in 1950 but only 16% in 2000. During the same time period, forest fragments less than 10 km^2^ in area increased from 5% of the total forest area to 24%. Considering that more than 90% of the Malagasy fauna inhabits forests and woodlands, such massive habitat destruction and fragmentation inevitably imperils Malagasy fauna [30].

**Fig 1:**
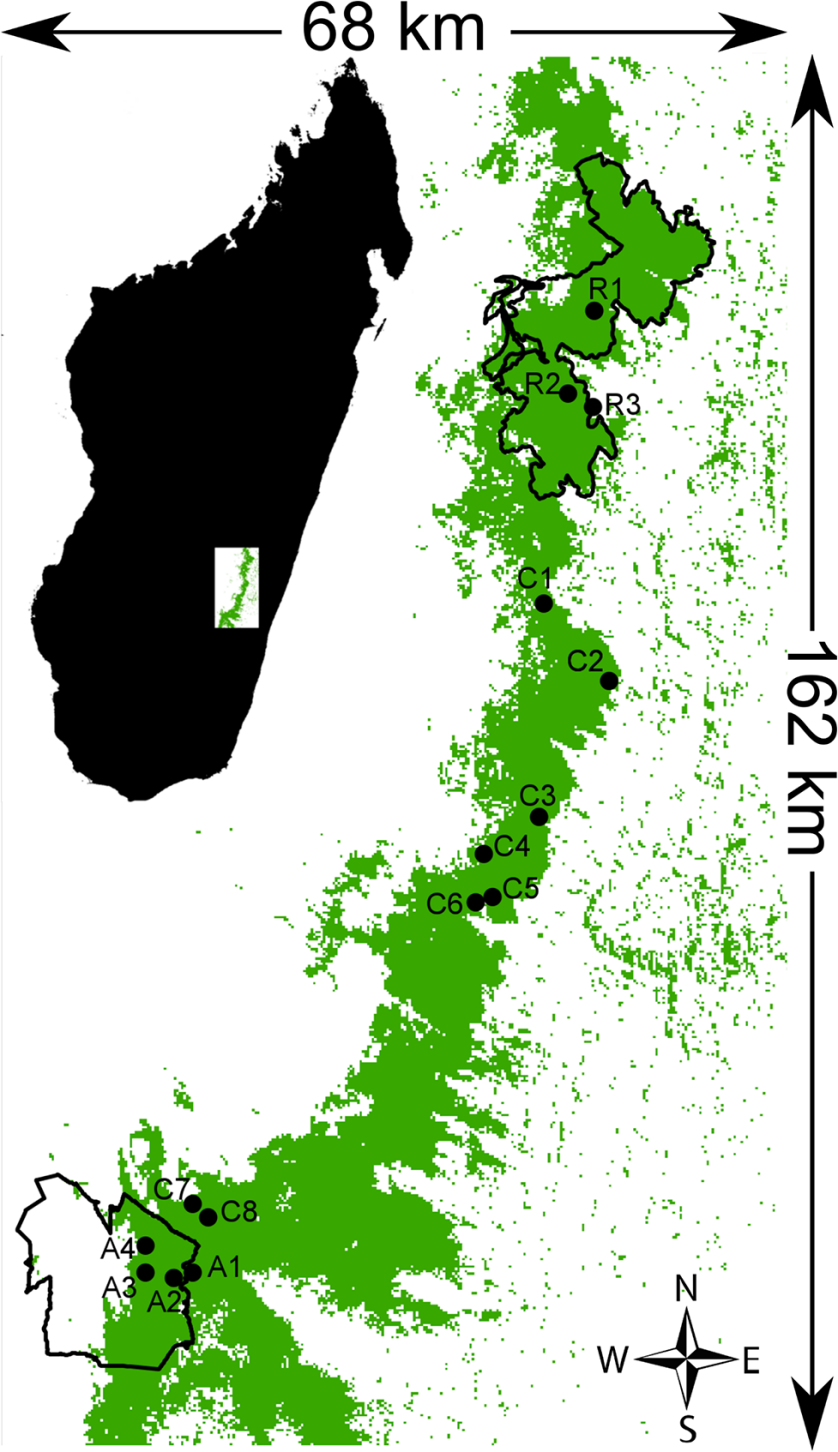
Map of the study area with the locations of the 15 sampling sites. Intact and degraded forests have been pooled here into just one forest type shown by the green color. White shows non-forest areas. Thick black lines show the boundary of the Ranomafana National Park (North) and Andringitra National Park (South). Forest cover modified from Hansen et al. [38]. Site coordinates from Goodman and Razafindratsita [37].

The peril of Malagasy biodiversity is well known. During the World Park Congress in 2003, the former president of Madagascar, Marc Ravalomanana, publicized the goal of tripling the total size of protected areas to cover 10% of the country [32]. In this context, the Ambositra-Vondrozo Corridor (COFAV) was established in the south-eastern part of Madagascar (Fig 1), within an area of high conservation value [33]. The corridor stretches for more than 200 km with a width of 2 to 50 km and covers 1,352 km^2^ [34]. Apart from adding more protected habitat, the corridor’s principal objective was to connect two major national parks, Ranomafana and Andringitra, which are 95 km apart, have the areas of 416 and 311 km^2^, and are located at elevations from 500 to 1380 and from 650 to 2650 m above sea level, respectively. The forest corridor is protected at the level VI of the International Union for Conservation of Nature (IUCN) categories, while the parks are at the level II. These two parks have been subject to intensive scientific research even prior to their establishment and until today [35–37].

Here, we combine detailed forest cover data, published survey data on vertebrates and a simulation model to assess the current quality of the Ambositra-Vondrozo Corridor and its influence on the distribution and dynamics of species outside and within the two national parks. We analyze data on birds, amphibians, reptiles, small mammals and lemurs, sampled in the Ranomafana NP, Andringitra NP and the corridor, as reported by Goodman and Razafindratsita [37].We test whether species richness is different in the national parks and in the corridor, analyze possible differences in community composition, and use statistical models to analyze whether forest cover, altitude and other environmental variables explain species richness. Finally, we use a stochastic simulation model to examine the effectiveness of the corridor in allowing dispersal between the two parks at present and under scenarios of further forest loss.

## Material and Methods

### Species data

We used the survey data reported by Goodman and Razafindratsita [37]. They sampled altogether 15 study sites during two time periods: four sites in the Andringitra NP in 1993, three sites in the Ranomafana NP, and eight sites in the corridor (Fig 1) in 1999 and 2000. We analyzed the data for five taxa, including 101 species of birds, 106 species of amphibians, 53 species of reptiles, 28 species of small mammals and 12 species of lemurs. The sampling methods and procedures used for each taxon were the same at each study site. Point counts and mist nets were used for birds, pitfall traps and systematic search of potential habitat (e.g. dead wood, palm tree) for amphibians and reptiles, and standard traps (Sherman and National) and pitfalls were used for small mammals. Lemurs were surveyed along transects. Observations on additional species during the sampling periods were added to the data. The sampling effort varied slightly from one site to another, but as species accumulation curves mostly saturated [37], we did not consider it necessary to use any correction for the sampling effort.

### Habitat description

**Fig 2:**
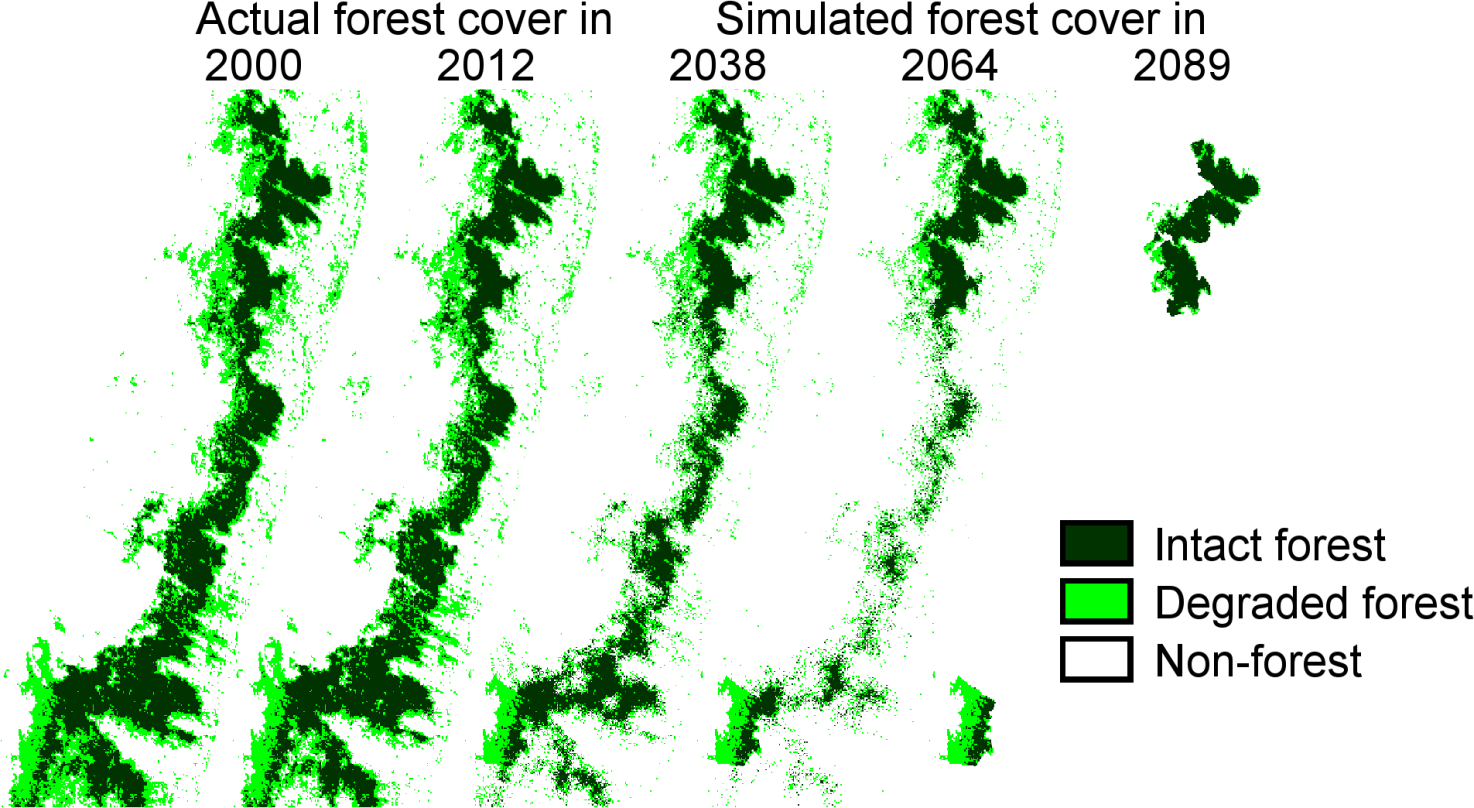
The observed forest cover in 2000 and 2012 and the predicted forest cover in 2038, 2064 and 2089. The rate of deforestation that was observed between 2000 and 2012 was assumed to continue to the future. The resolution of the map is 285 x 285 m. Black and green colors indicate intact and degraded forest, respectively.

Forest cover for the study area was obtained from Hansen et al. [38], who provide a high resolution map (1 arcsecond ≈ 28.5m x 28.5m) for the percentage of tree cover in 2000, as well as comparable data for forest loss and gain from 2000 until 2012, based on Landsat 7 ETM + imagery. For our modeling and statistical analyses (below), we generated maps for 2000 and 2012, with habitat classified into three classes, intact forest with tree cover >96%; degraded forest with tree cover between 68% and 96%; and the rest, which is considered to be non-forest (Fig 2). These threshold values were selected to give the best match with the forest classification of Puhakka [39], which combines satellite images with ground truthing.

The simulation model, described below, was run for the 2000 and 2012 forest maps as well as for a scenario assuming continuation of deforestation as observed between 2000 and 2012 (Fig 2). Deforestation was assumed to propagate from existing non-forest cells and to depend on altitude. A description of the deforestation algorithm is presented in S1 File.

### Data analysis

We classified the sampling sites into three groups, Andringitra NP (A), Ranomafana NP (R) and the corridor (C). Sites C7 and C8 are located outside the boundaries of the Andringitra NP, but due to their close proximity to the park (see Fig 1) we included them in group A. Group C thus consists of sites C1 to C6.

We first examined the data using descriptive statistics. Species richness was recorded for individual sites as well as the average for the three groups of sites. For each site, we calculated average body mass or body length of the species, depending on which trait was available. Species traits were obtained from Glaw and Vences [40], Goodman and Razafindratsita [37], Goodman and Benstead [41], Sinclair and Langrand [42], and the databases www.birdlife.org, http://amphibiaweb.org, http://www.reptile-database.org and http://www.iucnredlist.org. We ran a principal component analysis (PCA) after the data were transformed using the Hellinger distance [43] to compare communities among sites. In addition, we used Poisson regression to investigate whether differences in species richness and body size within parks and in the corridor were related to environmental variables such as forest cover, temperature, elevation, and precipitation.

### Simulation model

To analyze the importance of the corridor in the dynamics and distribution of species, we constructed a spatially explicit, discrete-time stochastic patch occupancy model. The model is adapted from the one developed by Rybicki and Hanski [44]. The entire study area is covered by a grid of 571 x 313 (= 178 723) cells, where each cell is a square of 285 x 285 m as in Fig 2. This resolution was obtained by aggregating 10 x10 grid cells in the original map. The forest cover of each 285 x 285 m cell was calculated by averaging the values for the 10 x 10 constituent cells, and then classifying the aggregated cell into either intact forest, degraded forest or non-forest as explained above.

In the simulations, we modeled the dynamics of generalist species and species specialized to either intact or degraded forests. For every species, we define a fitness *q* with respect to the forest type of the cell. *Intact forest specialists* have their maximal fitness *q* = 1 in cells classified as intact forest whereas the other cell types are inhospitable (*q* = 0) for them. Similarly, *degraded forest specialists* have *q* = 1 in degraded forest cells and *q* = 0 elsewhere. The *generalists* have fitness *q* = 0.5 in both intact and degraded forest and *q* = 0 elsewhere. Thus all species have zero fitness in non-forest cells. In addition to fitness, each species is characterized by its colonization, extinction, and dispersal parameters, *c*, *e*, and *a*, respectively, which are described below.

The dynamics of a species is given by a Markov chain in which time proceeds in discrete steps *t* = 0, 1, 2,… At every time step from *t* to *t*+1, there are two possible state transitions for each cell, extinction (an occupied cell becomes unoccupied) and colonization (an unoccupied cell becomes occupied). The probability that an occupied cell *i* goes extinct is *1- exp*(*-e/q*(*i*)), where *q*(*i*) > 0 is the fitness in cell *i*. In the case *q*(*i*) = 0, the cell is uninhabitable and therefore extinction is immediate (with probability 1). The probability that an unoccupied cell *i* with *q*(*i*) > 0 is colonized at time step from *t* to *t*+1 is 1-*exp*(-*cS*(*i*, *t*)), where *S*(*i*,*t*) is the connectivity of cell *i* at time *t* (below).

**Fig 3:**
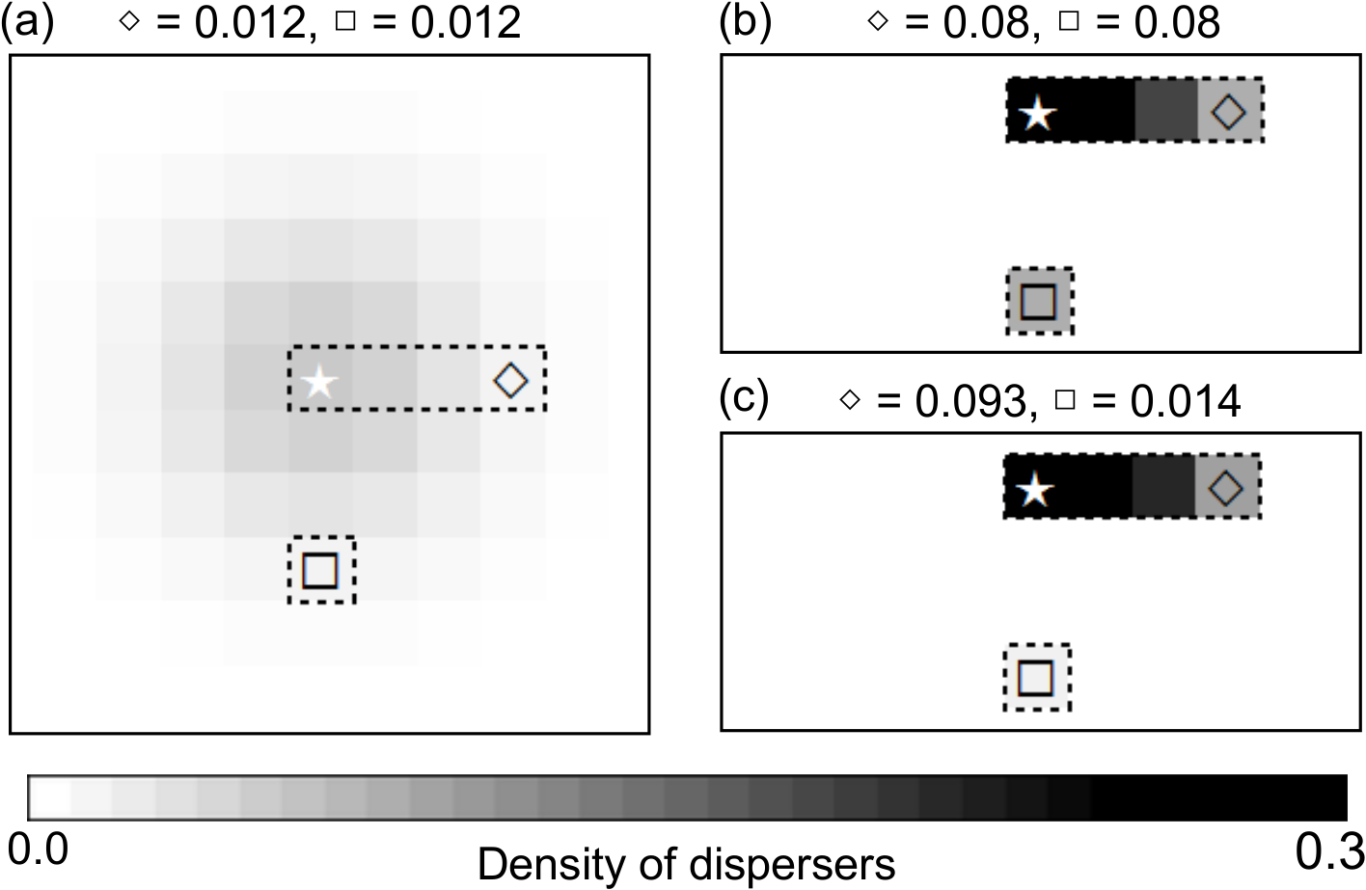
Proportion of dispersers reaching different cells for three modes of dispersal in a simple hypothetical situation. Panels (a), (b) and (c) are for passive dispersal, active dispersal without gap-avoidance, and active dispersal with gap-avoidance, respectively. Suitable habitat (*q* = 1) occurs within the dashed rectangles, whereas the remaining habitat is unsuitable (*q* = 0). The only source of propagules is the cell with a white star. The numbers above the maps give the proportion of propagules reaching the cells indicated by an empty diamond and square, which are located at equal distances from the source.

We next define three types of connectivity measures corresponding to three modes of dispersal: passive, active without gap-avoidance, and active with gap-avoidance (Fig 3). All modes use a 2-dimensional Gaussian dispersal kernel.

In passive dispersal, connectivity of cell *i* at time *t* is defined as

$$S_{1}(i,t)=\sum_{j}O(j,t)q(j)f(i,j),$$ (1)

where $f(i,j)=\frac{1}{2\pi a^{2}}exp\left(-\frac{r_{ij}^{2}}{2a^{2}}\right)$ is the Gaussian dispersal kernel with length scale parameter *a*, and *r*_*ij*_ is the two-dimensional Euclidian distance between cells *i* and *j*. *O*(*j*,*t*) is an indicator variable with the value of 1 if cell *j* is occupied at time *t* and 0 otherwise.

For active dispersal without gap-avoidance, we assume that the colonization probability increases with the fitness of the species in the target cell; thus dispersers never move to non-forest cells. In this case, connectivity is defined as

$$S_{2}(i,t)=\sum_{j}O(j,t)q(j)\frac{f(i,j)q(i)}{\sum_{k}f(k,j)q(k)}.$$ (2)

The term $\frac{f(i,j)q(i)}{\sum_{k}f(k,j)q(k)}$ represents the fraction of offspring dispersing from the source cell *j* to the target cell *i*.

Finally, we consider active dispersal with gap-avoidance. In this case, connectivity is given by a sequence of small-scale active dispersal. We start by computing the initial connectivity,

$$S_{3}^{1}(i,t)=\sum_{j}O(j,t)q(j)\frac{f_{s}(i,j)q(i)}{\sum_{k}f_{s}(k,j)q(k)},$$ (3)

where *f*_*s*_ is the Gaussian kernel with a small length scale parameter *a*_*s*_ *≪ a*. The initial connectivity $S_{3}^{1}$ can be interpreted as the distribution of propagules after a single movement step. The distribution of propagules after *n* movement steps is defined recursively as

$$S_{3}^{n}(i,t)=\sum_{j}S_{3}^{n-1}(j,t)\frac{f_{s}(i,j)q(i)}{\sum_{k}f_{s}(k,j)q(k)}.$$ (4)

If the landscape consists only of suitable cells, connectivity $S_{3}^{n}$ after the *n* ^*th*^ step corresponds to the connectivities *S*_*1*_ and *S*_*2*_ with length scale parameter $a_{s}\sqrt{n}$. In our calculations, we assumed that *a*_*s*_ *=* 1 and chose the number of iterations *n* so that in a uniform landscape $S_{1}=S_{2}=S_{3}^{n}$. If the landscape is not uniform, calculating the connectivity in this manner constrains a species to disperse through suitable neighboring cells and hence avoid crossing gaps.

We modeled scenarios without and with environmental stochasticity. In the presence of environmental stochasticity, we assumed that the probability of extinction in occupied cells is given by $1-exp\left(-\frac{e}{q(i)E(i,t)}\right),$ where *E*(*i*,*t*) represents the environmental conditions at site *i* at time *t*. We assumed that *E*(*i*,*t*) follows log-normal distribution with mean zero and variance *σ* ^2^. The aim of including environmental stochasticity was to increase the extinction risk due to unfavorable environmental conditions in year *t*, and we hence truncated the distribution of *E*(*i*,*t*) by setting values above one to the value of one. We controlled for spatial correlation in environmental stochasticity by a parameter *w*. At *w* → 0 the noise is spatially uncorrelated (the values of *E*(*i*,*t*) are independent between the sites), at *w* → ∞ stochasticity is globally correlated (all sites have the same value for *E*(*i*,*t*) in a given year), whereas intermediate values of *w* correspond to regional stochasticity, i.e. spatially correlated environmental stochasticity (sites close to each other have more similar values for *E*(*i*,*t*) than sites far away from each other). For technical details of how regional stochasticity was implemented see S2 File and [44, 45]. One example of regional stochasticity is given by cyclones, which hit Madagascar on average 1.4 times per year [46], and which are known to increase the mortality rate of forest inhabiting fauna and flora [47].

We analyzed how landscape structure (Fig 2) influences the number of occupied cells at a quasi-stationary state, referred to as equilibrium below, as well as the transient dynamics. For each set of parameters, we run at least three replicates. The equilibrium simulations were initialized with all cells occupied. We calculated patch occupancy at equilibrium as the average number of occupied cells during time steps from 250 to 500, as by this time the species had reached the quasi-stationary state. We analyzed transient dynamics by having the species initially present (with full occupancy) only in the northern or the southern park, and then calculating the time until the initially unoccupied park became colonized for the first time. We call this transient time. Transient time is relevant for recolonization as well as for assessing the rate of gene flow between the parks.

## Results

### Deforestation in the corridor

The amounts of intact and degraded forest lost during the 12 year period from the year 2000 to the year 2012 were 140 and 290 km^2^, which make 10.6% and 18.9% of the intact and degraded forest area in 2000, respectively. The annual rate of forest loss is thus 0.88% for intact forest and 1.5% for degraded forest.

At present, the forest corridor is very narrow near Andrambovato (site C1) and 16.5 km south of Ankopakopaka (site C6). Our deforestation simulations, illustrated in Fig 2 at 28 year intervals and in S1 Fig at 5 year intervals, suggest that the corridor will be first broken in these locations. Assuming that deforestation proceeds at the same rate as between 2000 and 2012, the corridor will be broken up into five relatively large blocks after 30 years. After 40 years these blocks become very isolated (up to 10 km), and only a few patches of degraded forest will connect the blocks and the national parks. Very little forest remains between the two parks after 60 years, and the corridor will disappear completely after about 80 years.

### Patterns of species richness

**Fig 4:**
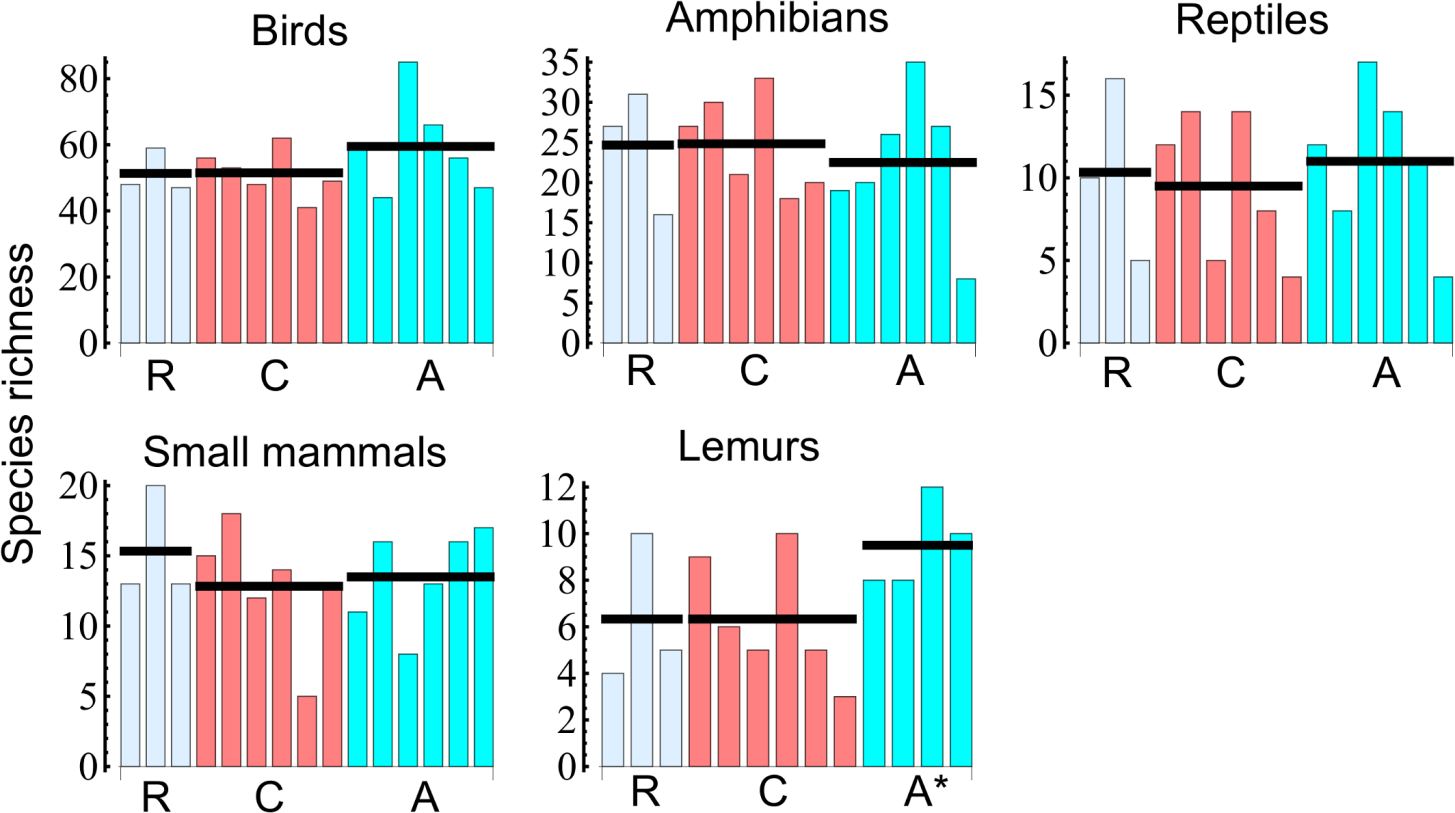
Species richness per site (vertical bars) and the average species richness per region (horizontal bars) for different taxa. The group R (light blue) includes sites in the Ranomafana National Park, the group C (pink) the corridor sites without Manambolo 1 and 2 (see Fig 1), and the group A (cyan) includes Manambolo 1, 2, and the sites within the Andringitra National Park. For lemurs, A* denotes Manambolo 1, 2 and two sites in Andringitra.

Before 2000, the average species richness was roughly the same in the two parks and the corridor (Fig 4). Ranomafana NP has slightly more species of small mammals, whereas lemur species richness is highest in Andringitra NP. Fewer species of small mammals and reptiles are found in the corridor than in the national parks, but overall there is no substantial difference in species richness between the parks and the corridor.

The numbers of species endemic to Ranomafana NP, Andringitra NP, and the corridor were generally low and similar for each of the three regions (Fig A in S2 Fig). Furthermore, a large fraction of species scored as endemic to one of the regions may have just remained undetected in the two other regions. The number of species present in all three regions is high for birds, small mammals and lemurs, indicating that the majority of species have continuous distributions across the entire region. Additional regression analyses on the effects of environmental variables on species richness and body size, as well as ordination analyses, showed no significant differences between the parks and the corridor. These results are reported in S2 Fig and S3 File.

### Model-predicted equilibrium occupancy

We used the stochastic patch occupancy model to examine how the fraction of suitable habitat cells that were occupied in the two parks depended on the corridor and the mode and spatial scale of dispersal. In the simulations, we fixed the habitat suitability map to that observed either in year 2000 or in year 2012, or to the simulated projected forest cover for the years 2038, 2064 or 2089 (Fig 2).

**Fig 5:**
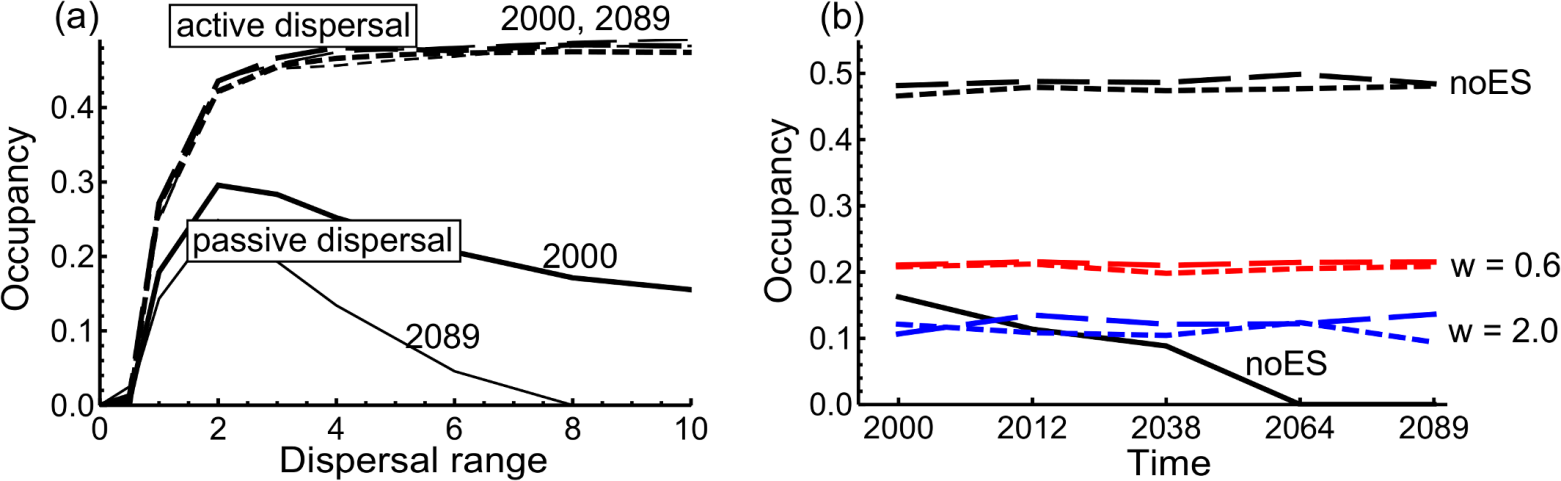
Average equilibrium occupancy of intact forest specialists in the Andringitra park. The vertical axis gives the fraction of occupied cells out of the suitable ones as an average for the last 250 time steps of the 500 steps long simulation. In both panels, solid, long-dashed, and small-dashed lines are for the passive, active without gap-avoidance, and active with gap-avoidance dispersal, respectively. Panel (a) shows the change in occupancy as a function of the spatial scale of dispersal. Thick and thin lines represent the result for the years 2000 and 2089. Panel (b) shows simulated equilibrium occupancies for observed and simulated forest cover maps shown in Fig 2. Black lines show the results without environmental stochasticity. Red (*w* = 0.6, *μ* = 0, *σ* ^2^ = 1) and blue (with *w* = 2, *μ* = 0, *σ* ^2^ = 1) lines show the results with two spatial scales of regional stochasticity, for which cases the species with passive dispersal went always extinct. Species’ parameters were set to *c* = 0.2, *e* = 0.1, *a* = 10.

We summarize the main results on the equilibrium abundances in the parks in Fig 5. First, the spatial scale of dispersal has contrasting effects depending on the mode of dispersal (Fig 5A). With active dispersal with or without gap-avoidance, longer dispersal distances lead to higher abundances, and the difference in the quality of the corridor between 2000 and 2089 makes little difference. In contrast, with passive dispersal, abundance is maximized at intermediate range of dispersal. As dispersal range increases, abundance decreases, and the difference in the quality of the corridor between 2000 and 2089 has a very large effect for species with long-range dispersal.

The quality of the corridor has little influence on the abundance of active dispersers in the parks, even if the corridor would disappear completely, as happens in our simulations by 2089 (Fig 5B). In contrast, for passive dispersers the abundance of intact forest specialists in the parks decreases as corridor destruction proceeds (Fig 5B). For the same values of the colonization and extinction parameters used in Fig 5B, degraded forest specialists have similar occupancy as the intact forest specialists whereas the generalists always go extinct (S3 Fig). A generalist may persist if the colonization rate is very high, in which case the response to corridor destruction is qualitatively similar to the response of the specialist species. Hence, if the colonization (and turnover) rate is higher than in the above examples, the destruction of the corridor has less effect (S3 Fig).

We next added to the model environmental stochasticity, which reduced the predicted abundances in each scenario (Fig 5B). Using the parameters of Fig 5B, passive dispersers had low abundance already without environmental stochasticity, and they went extinct when environmental stochasticity was included in the model. For active dispersers, abundances decrease as the spatial scale of regional stochasticity increases, but the quality of the corridor has little effect on the occupancy in the parks (Fig 5B).

### Model-predicted transient dynamics

**Fig 6:**
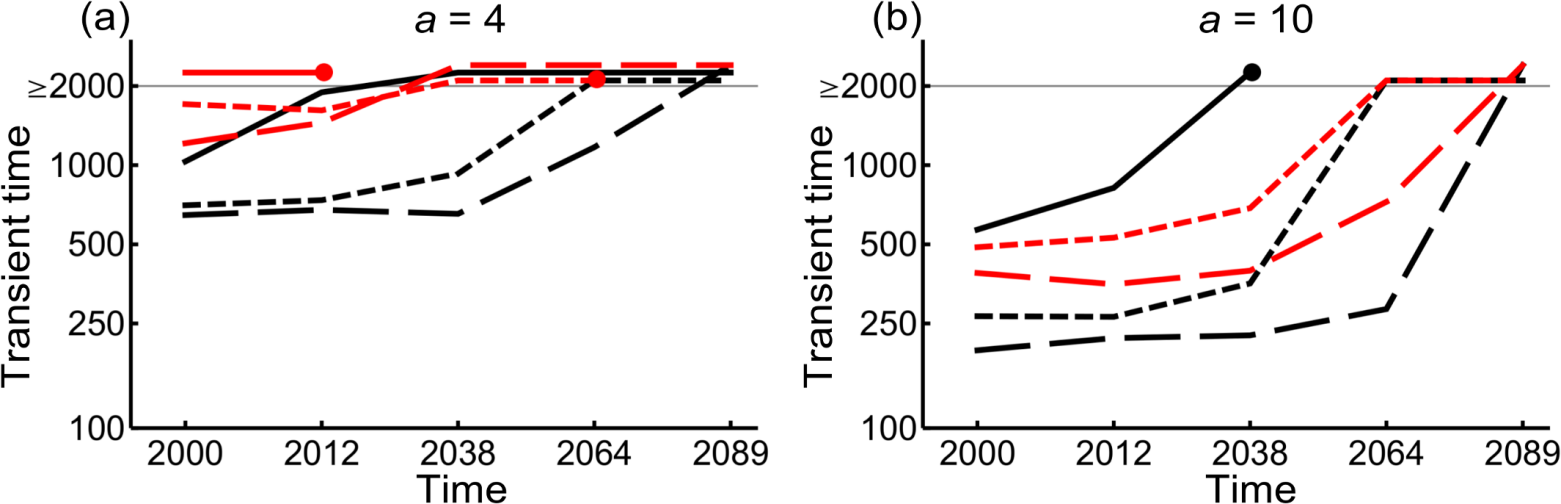
Transient time to reach the Ranomafana park when only the Andringitra park is initially occupied. Black (red) lines show intact forest specialists without (with) regional stochasticity. Line types are as in Fig 5. Lines above ≥2000 (gray line) mean that the species persisted during the entire simulation period of 2000 years but did not reach the Ranomafana park. Lines that end with dots mean that the species went extinct for further loss of forest cover. The spatial scale of dispersal is *a* = 4 in panel (a) and *a* = 10 in panel (b). Colonization and extinction rate parameters are *c* = 0.2 and *e* = 0.1, and parameters for regional stochasticity are *μ* = 0, *σ* ^2^ = 1 and *w* = 1.

As expected, active dispersal always leads to a shorter transient time than passive dispersal (Fig 6), partly because occupancy is higher when dispersal is active but also because active dispersal enhances the colonization of isolated fragments of habitat (Fig 3). Because the colonization of isolated fragments of suitable habitat is reduced for dispersal with gap-avoidance, the transient time under that mode of dispersal is longer than in active dispersal without gap-avoidance. From 2038 onwards, the colonization ability of intact forest specialists declines because the majority of intact forests have become converted into degraded forests or non-forest habitats (Fig 6). For active dispersers with gap-avoidance, the quality of the corridor in 2064 is too poor to allow the intact forest specialists to reach the other park. In general, there is no slowing down of colonization for active dispersers without gap-avoidance until 2064. For the two other modes of dispersal, transient times become longer as the corridor becomes more degraded. For intact forest specialists with passive dispersal, the destruction of the corridor between 2000 and 2012 greatly reduced functional connectivity between the two parks (Fig 6). The contrast between active and passive dispersers is evident for low and relatively low colonization rates, while with high colonization rate the transient time is approximately equal for all modes of dispersal (Fig C in S4 Fig). Smaller values for dispersal parameter (*a <* 4) would further increase transient time for all modes of dispersal (Fig C in S4 Fig). If dispersal range is large enough, passive dispersers go extinct (Fig 5A) whereas active dispersers without gap-avoidance colonize the other park after one time step—only in that case would transient time be invariant to complete corridor destruction (Fig E in S4 Fig).

We found that environmental stochasticity increases the transient time several fold compared to the model without environmental stochasticity because spreading towards the other park is now slowed down due to more frequent extinctions. With environmental stochasticity, small to intermediate correlation in the spatial scale has little influence on transient time; for strong spatial correlation, the transient time is longer (Fig F in S4 Fig). In general, the transient time for the degraded forest specialists is longer than that for the intact forest specialists, except when the amount of continuous intact forest is greatly reduced in the corridor i.e. after 2038 (S4 Fig).

## Discussion

Corridors remain one of the most popular approaches to biodiversity conservation [4]. Unfortunately, though corridors are conceptually appealing, it remains difficult to obtain robust evidence about increased viability of fragmented populations due to corridors in natural systems [8, 48]. In principle, empirical evidence could be obtained by measuring gene flow and changes in species abundances in core areas with dissimilar structural and functional connectivities. In practice, however, this is difficult or even impractical to do in the case of large real-world corridors, because of the high cost of collecting the data and slow dynamics at large spatial scales [48].

The presence of individuals in the corridor has commonly been used as the response variable while assessing the significance of corridors, though the mere presence does not allow conclusive inference about the functionality of the corridor [8, 48]. Nonetheless, at large spatial scales the presence of individuals in the corridor is a useful necessary condition for corridor functionality. In the present study, the similarity of the species composition in the two national parks and the forest corridor suggests that the corridor consists of favorable habitat for the vertebrate species that we have analyzed. Moreover, our analyses indicated no difference in species richness between the parks and the corridor in samples obtained with similar effort, which suggests that there is a sufficient amount of high quality habitat in the corridor to support breeding populations. We hence conclude that the habitat in the corridor and in the parks was of similar quality at the time of the field work in 1993 to 2000.

Unfortunately, the situation has become worse since 2000, and it is becoming rapidly even worse. Based on the detailed mapping of the forest between 2000 and 2012, 36.5 km^2^ of forest (intact plus degraded) have been converted to non-forest habitat annually in the corridor, which makes 1.2% per year. At present, the width of the corridor is generally much more than 1 km, which is considered to be sufficient to facilitate vertebrate movements ([49] and references therein). However, the corridor is already very narrow with the width of 1 km near Andrambovato (about 10 km south from Ranomafana), and gaps are already appearing in this narrow band. If deforestation continues with the same rate into the future, as we have assumed in our simulations, the situation becomes quickly worse especially for the intact forest. Our model indicates that by 2040 the corridor will be composed of five blocks of intact forest connected by small fragments of degraded forest.

We used a spatially realistic, grid-based presence-absence metapopulation model to investigate the functionality of the current corridor and the modified corridor with projected deforestation. Spatial models have been used to gain insight into how landscape quality influences population dynamics [50]. In general, such models aim to include many details and high-quality demographic data are required for parameter estimation. As we could not parameterize the model for any particular species, we focused on examining the effect of the mode of dispersal—passive, active without gap-avoidance, and active with gap-avoidance—on the predicted abundance of the species and their capacity to move through the corridor. Our implementation of these modes of dispersal is necessarily simplified and most species are not likely to strictly conform to these modes. For example, while many species of plants and fungi have passive airborne dispersal, the same species can also be dispersed by actively moving animals, leading to a mixture of passive and active modes of dispersal. For another example, we assumed that gap size does not make a difference for active dispersers, which clearly is a simplified assumption. However, even if real dispersal behaviors are likely to fall between the simplified behaviors considered here, they are nonetheless informative in providing general insight to the influence of the type of dispersal.

The forest habitat was divided into intact and degraded forest, and we modelled the respective specialist species as well as forest generalists. One example of an intact forest specialist is the black-and-white ruffed lemur (*Varecia variegata*), which is the largest lemur in the region and classified as Critically Endangered in the IUCN red list [51]. The greater bamboo lemur (*Prolemur simus*) is a bamboo specialist and one of the most critically endangered primates in the world [51, 52]. Bamboos are most abundant in degraded forests, and hence the greater bamboo lemur is best classified as a degraded forest specialist. One example of a generalist that is present both in degraded and intact forests is the brown mouse lemur (*Microcebus rufus*), with somewhat higher abundance in degraded forests [53]. This species is the smallest lemur in the region, and it is listed as Vulnerable in the IUCN red list [51]. The view that not only the intact forests but also the degraded forests are important in supporting biodiversity was highlighted by a recent meta-analysis, which reported a high level of biodiversity in degraded (selectively logged) forests throughout the tropical regions [54].

We found a major difference in the dynamics of actively versus passively dispersing species. In the case of the former species, degradation of the corridor does not make much difference to the abundance of the species in the parks, assuming that the forest in the parks does not deteriorate; the parks are large enough to support viable populations on the medium term. However, there are four caveats to this conclusion. First, adding regional stochasticity, which ubiquitous in nature, increases the risk of extinction of even large populations, and hence increases the risk of species extinction in the national parks. In this situation, populations in the corridor may become essential for recolonization and hence for long-term persistence. Second, many previous studies have demonstrated that mammals and birds have significant extinction risk even in large parks [55] due to hunting and poaching, which pose a severe threat in many tropical parks [56–58]. In Madagascar, national parks are additionally under threat due to illegal mining and selective logging [59, 60]. Third, our model assumes that ‘intact forest’ is uniformly suitable across the two parks and the corridor, whereas in reality many species are more specialized in their habitat selection, and have therefore smaller equilibrium abundances and are more vulnerable to extinction than our results would suggest. Fourth, the adverse effect of inbreeding and loss of genetic variation on population viability were not considered in our model.

The situation is worse for passively dispersing species, because in this case dispersing individuals do not select the habitat where they settle down and hence a fraction of dispersers are lost in the surrounding matrix. This will become a problem quickly if the deterioration of the corridor continues, especially in the case of species with long dispersal distances (several kilometers). This conclusion is consistent with many previous theoretical studies that have emphasized the importance of dispersal mortality for population viability [61, 62]. To make the simulation outcomes comparable, we assumed that each species produces the same number of propagules independently of the mode of dispersal, such assumption leads to lower prevalence for passive than active dispersers, as the former lose some of their propagules to the unsuitable matrix. As another point of comparison, we could have assumed that the passive dispersers have a higher rate of propagule production so that their prevalence would be initially the same as for active dispersers. While this assumption would increase the overall prevalence of the passively dispersing species, it would not change the qualitative result that passive dispersers are more vulnerable to fragmentation than active dispersers.

Louis and his coworkers have studied genetic diversity and gene flow in the black-and-white ruffed lemur in southeastern Madagascar [63–65]. They have concluded that there is a low level of gene flow between Ranomafana and other southern parks and special reserves and that populations are inbred even within the Ranomafana NP, probably due to past bottlenecks in population size [63–65]. Although we cannot make any quantitative predictions with our model, we can use the results as a baseline to assess how gene flow would be further reduced with continued destruction of the corridor. The steep increase in the transient time, corresponding to a significant decline in connectivity and gene flow, in the case of active dispersers with gap-avoidance after 2040 will significantly exacerbate the adverse effects of genetic isolation. At present, genetic rescue and recolonization are still possible but further reduction of the corridor will necessarily reduce these possibilities. Although the empirical genetic study was concerned with a single species of lemur, the result is likely to apply to a wide range of taxa, many of which have lower dispersal propensity than large lemurs.

In conclusion, the width and the total area of the forest corridor are large enough at present to support viable populations, to facilitate movements and to compensate for the adverse effects of regional stochasticity and genetic isolation. However, given that the corridor is already starting to become fragmented, there is some urgency to implement more stringent measures of conservation to prevent continuing deforestation. Not only does high-quality corridor enhance functional connectivity but, as we have concluded from empirical and modelling analyses, is a valuable reservoir of populations and genetic diversity. Many species occur only at certain altitudes, and any reduction in the corridor may have especially adverse consequences on their occurrence. Some studies have highlighted possible negative effects of corridors, for instance as routes along which infectious diseases and invasive species may spread [7, 66]. Such risks are small in the case of the forest corridor in Madagascar, which has existed for millennia, and where the question is about protecting what has existed rather than about creating something new.

## Supporting Information

S1 Fig Predicted forest cover in 5 year intervals for the state of the corridor between 2012 and 2089. (PDF)

S2 Fig Results of the statistical analyses of community structure, species richness and body size. Fig A in S2 Fig shows the number of species unique to Ranomafana NP (R), Corridor (C), and Andringitra NP (A). RA denotes the number of common species to Ranomafana NP and Andringitra NP. RCA denotes the number of species present across the entire landscape. Fig B in S2 Fig shows the Poisson regression of species richness per site as a function of average temperature during the survey. Dashed (solid) lines with up-ward (down-ward) pointing triangles show the data for sites above (below) 1000 m asl. Blue, red and cyan denote sites in the Ranomafana park, the corridor and the Andringitra park, respectively. Empty symbol indicates missing temperature data that were extrapolated using the nearest site for visualization. Model comparisons and *P* values are in S3 File. Fig C in S2 Fig shows the effect of temperature (mT), altitude above and below 1000m asl (altC), and whether the site belongs to the corridor or not (PC) on the logarithm of body size of lemurs using Poisson regression. Red (indigo) fitted lines are for sites in corridor (park). Solid (dashed) lines are for sites below (above) 1000 m. The symbols are as in Fig B in S2 Fig. Figs D-H in S2 Fig show the results of the principal component analysis for each taxonomic group.
(PDF)

S3 Fig Complementary results on equilibrium abundance for different parameter values. S3 Fig shows equilibrium occupancy for both Ranomafana NP and Andringitra NP for intact forest specialists (blue), degraded forest specialists (red), and generalists (black). Solid, dashed and dotted lines represent, passive dispersal, active without gap-avoidance and with gap-avoidance, respectively. Error bars show minimum and maximum value for different replicates. In Figs A-B in S3 Fig, *c* = 0.2, *e* = 0.1, *μ* = 0, *σ* ^2^ = 1. Figs C-D in S3 Fig show the outcome of the simulation for larger values of colonization and extinction rates without regional stochasticity.
(PDF)

S4 Fig Complementary results on transient dynamics for different parameter values. The full result for Fig 6 is shown in Fig A in S4 Fig, which includes degraded forest specialists and forest generalists. Figs B-E in S4 Fig show the influence of high values of colonization and extinction rates, and dispersal range on transient time without regional stochasticity setting initial occupancy in Ranomafana NP or Andringitra NP. Intact forest specialists, degraded forest specialists, and generalists are represented by blue, red, and black, respectively. Solid, dashed and dotted lines represent, passive dispersal, active without gap-avoidance and with gap-avoidance, respectively. Lines on the ≥2000-mark mean that the other park was never reached after 2000 time steps. Solid dots, empty dots, and empty squares above Ext (upper gray line) mean that the passive, active without gap-avoidance, and active with gap-avoidance dispersers went extinct. The figures show that higher values of colonization and extinction rates reduce the difference among species and among modes of dispersal. Fig E in S4 Fig shows that transient time becomes independent of corridor quality for extremely large mean dispersal. Fig F in S4 Fig shows the influence of the correlation in the spatial scale in environmental stochasticity (*w*) on transient time. Black represents models without regional stochasticity. Green, red, and blue represent model with regional stochasticity with *w* = 0.1, 1, and 2, respectively. Fig F in S4 Fig shows that including regional stochasticity leads to higher transient time. Small to moderate value of *w* yield similar transient times; very large *w* however lengthens transient time.
(PDF)

S1 File Detailed description of the deforestation algorithm. (DOCX)

S2 File Environmental stochasticity in the simulation model. (PDF)

S3 File Statistical analyses if community composition and species richness in relation to environmental variables. (DOCX)

S4 File Supporting text for running the simulations. (DOCX)

## References

[1] Millennium Ecosystem Assessment. Ecosystem and Human Well-being: Biodiversity Synthesis. 2005.

[2] IUCN. The Durban Action Plan. One of the outputs to the 2003, 5th IUCN World Parks Congressheld in Durban, South Africa. 2003.

[3] Jenkins CN, Joppa L. Expansion of the global terrestrial protected area system. Biological Conservation. 2009;142(10):2166–74.

[4] CBD. CoP 10 decision X/2. Strategic Plan for biodiversity 2011–2020. 2010.

[5] MacArthur RH, Wilson EO, editors. The Theory of Island Biogeography. Princenton N.J.: Princeton University Press; 1967.

[6] Hanski I, Gaggiotti OE, editors. Ecology, Genetics, and Evolution of Metapopulation. Amsterdam: Elsevier; 2004.

[7] Simberloff D, Farr JA, Cox J, Mehlman DW. Movement corridors—conservation bargains or poor investments. Conservation Biology. 1992;6(4):493–504.

[8] Beier P, Noss RF. Do habitat corridors provide connectivity? Conservation Biology. 1998;12(6):1241–52.

[9] Niemela J. The Utility of Movement Corridors in Forested Landscapes. Scand J Forest Res. 2001;16(sup003):70–8.

[10] Bennett AF. Linkages in the Landscape The Role of Corridors and Connectivity in Wildlife Conservation: IUCN; 1999.

[11] Beier P. Dispersal of Juvenile Cougars in Fragmented Habitat. The Journal of Wildlife Management. 1995;59(2):228–37.

[12] Haddad NM. Corridor use predicted from behaviors at habitat boundaries. American Naturalist. 1999;153(2):215–27. 10.1086/303163 29578761

[13] Haddad NM, Bowne DR, Cunningham A, Danielson BJ, Levey DJ, Sargent S, et al Corridor use by diverse taxa. Ecology. 2003;84(3):609–15.

[14] Castellon TD, Sieving KE. An Experimental Test of Matrix Permeability and Corridor Use by an Endemic Understory Bird. Conservation Biology. 2006;20(1):135–45. 16909666 10.1111/j.1523-1739.2006.00332.x

[15] Gillies CS, Clair CCS. Riparian corridors enhance movement of a forest specialist bird in fragmented tropical forest. Proceedings of the National Academy of Sciences of the United States of America. 2008;105(50):19774–9. 10.1073/pnas.0803530105 19017794 PMC2604990

[16] Forney KA, Gilpin ME. Spatial Structure and Population Extinction: A Study with Drosophila Flies. Conservation Biology. 1989;3(1):45–51.

[17] Gonzalez A, Lawton JH, Gilbert FS, Blackburn TM, Evans-Freke I. Metapopulation Dynamics, Abundance, and Distribution in a Microecosystem. Science. 1998;281(5385):2045–7. 9748167 10.1126/science.281.5385.2045

[18] Damschen EI, Haddad NM, Orrock JL, Tewksbury JJ, Levey DJ. Corridors increase plant species richness at large scales. Science. 2006;313(5791):1284–6. 16946070 10.1126/science.1130098

[19] Gilbert-Norton L, Wilson R, Stevens JR, Beard KH. A meta-analytic review of corridor effectiveness. Conservation biology: the journal of the Society for Conservation Biology. 2010;24(3):660–8. . 20184653 10.1111/j.1523-1739.2010.01450.x

[20] Hanski I, editor. Metapopulation Ecology Oxford University Press, USA; 1999.

[21] Baranga J. Kibale Forest game corridor: man or wildlife? In: Saunders DA, Hobbs RJ, editors. Nature Conservation 2: The Role of Corridors Surrey Beatty & Sons: Chipping Norton, New South Wales; 1991. p. 371–75.

[22] Dixon JD, Oli MK, Wooten MC, Eason TH, McCown JW, Paetkau D. Effectiveness of a Regional Corridor in Connecting Two Florida Black Bear Populations. Conservation Biology. 2006;20(1):155–62. 16909668 10.1111/j.1523-1739.2005.00292.x

[23] Algonquin to Adirondack. Algonquin to Adirondack home page 2014. Available: http://www.a2alink.org/. Accessed 28 October 2014.

[24] Southern Rockies Ecosystem Project. Southern Rockies Ecosystem Project home page 2014. Available: http://rockymountainwild.org/srep. Accessed 28 October 2014.

[25] Yellowstone to Yukon Conservation Initiative. Yellowstone to Yukon Conservation Initiative home page 2014. Available: http://y2y.net/. Accessed 28 October 2014.

[26] Linden H, Danilov PI, Gromtsev AN, Helle P, Ivanter EV, Kurhinen J. Large-scale forest corridors to connect the taiga fauna to Fennoscandia. Wildlife Biology. 2000;6(3):179–88.

[27] Myers N, Mittermeier RA, Mittermeier CG, da Fonseca GAB, Kent J. Biodiversity hotspots for conservation priorities. Nature. 2000;403(6772):853–8. 10706275 10.1038/35002501

[28] Gade DW. Deforestation and its effects in highland Madagascar. Mountain Research and Development. 1996:101–16.

[29] Lowry PP, Schatz G, Phillipson P. The classification of natural and anthropogenic vegetation in Madagascar. Natural change and human impact in Madagascar. 1997:93–123.

[30] Dufils J-M. Remaining forests cover In: Goodman SM, Benstead JP, editors. The natural history of Madagascar: Chicago, IL: The University of Chicago Press; 2003 p. 88–96.

[31] Harper GJ, Steininger MK, Tucker CJ, Juhn D, Hawkins F. Fifty years of deforestation and forest fragmentation in Madagascar. Environmental Conservation. 2007;34(4):325.

[32] Gouvernement Malgache. J. Off. Repub. Madagascar; 2004.

[33] Kremen C, Cameron A, Moilanen A, Phillips SJ, Thomas CD, Beentje H, et al Aligning conservation priorities across taxa in Madagascar with high-resolution planning tools. Science. 2008;320(5873):222–6. 10.1126/science.1155193 18403708

[34] Conservation International. Carbon Emissions Reduction Project in the Forest Corridor Ambositra-Vondrozo Forest Corridor (COFAV)–Madagascar. 2014 07 Jan 2014. Report No.: Contract No.: Version 1.0.

[35] Goodman SM. A floral and faunal inventory of the eastern slopes of the Réserve Naturelle Intégrale d'Andringitra, Madagascar: with reference to elevational variation: Field Museum of Natural History; 1996.

[36] Wright PC. The future of biodiversity in Madagascar: a view from Ranomafana National Park. Natural change and human impact in Madagascar. 1997:381–405.

[37] Goodman SM, Razafindratsita VR, editors. Inventaire biologique du Parc National de Ranomafana et du couloir forestier qui la relie au Parc National d'Andringitra: CIDST; 2001.

[38] Hansen MC, Potapov PV, Moore R, Hancher M, Turubanova SA, Tyukavina A, et al High-Resolution Global Maps of 21st-Century Forest Cover Change. Science. 2013;342(6160):850–3. 10.1126/science.1244693 24233722

[39] Puhakka E. Tropical rainforest corridor in Madagascar: satellite image-based land cover classification, landscape analysis and forest quality measurements [M.Sc. Thesis]: University of Helsinki; 2012.

[40] Glaw F, Vences M. A field guide to the amphibians and reptiles of Madagascar. 3rd edition ed: Vences & Glaw Verlag GbR; 2007. 496 p.

[41] Goodman SM, Benstead JP, editors. The Natural History of Madagascar: The University of Chicago Press; 2003.

[42] Sinclair I, Langrand O. Birds of the Indian Ocean islands: Random House Struik; 2013. 184 p.

[43] Legendre P, Gallagher ED. Ecologically meaningful transformations for ordination of species data. Oecologia. 2001;129(2):271–80. 28547606 10.1007/s004420100716

[44] Rybicki J, Hanski I. Species–area relationships and extinctions caused by habitat loss and fragmentation. Ecology Letters. 2013;16:27–38. 10.1111/ele.12065 23452159

[45] Gu W, Heikkilä R, Hanski I. Estimating the consequences of habitat fragmentation on extinction risk in dynamic landscapes. Landsc Ecol. 2002;17(8):699–710.

[46] Service de la Météorologie. Perturbations cycloniques à Madagascar de 1961 à 2000. Antananarivo: 2000.

[47] Dunham AE, Erhart EM, Wright PC. Global climate cycles and cyclones: consequences for rainfall patterns and lemur reproduction in southeastern Madagascar. Global Change Biology. 2011;17(1):219–27.

[48] Gregory AJ, Beier P. Response Variables for Evaluation of the Effectiveness of Conservation Corridors. Conservation Biology. 2014;28(3):689–95. 10.1111/cobi.12252 24606549

[49] Hilty JA, Lidicker WZ Jr, Merenlender A. Corridor ecology: the science and practice of linking landscapes for biodiversity conservation: Island Press; 2006. 344 p.

[50] Carroll C. Linking connectivity to viability: insights from spatially explicit population models of large carnivores In: Crooks KR, Sanjayan M, editors. Connectivity Conservation: Cambridge University Press; 2006 p. 369–89.

[51] IUCN. The IUCN Red List of Threatened Species. Version. 2014.3 2014. Available: http://www.iucnredlist.org. Accessed 10 May 2015.

[52] Wright PC, Johnson SE, Irwin MT, Jacobs R, Schlichting P, Lehman S, et al The crisis of the critically endangered greater bamboo lemur (Prolemur simus). Primate Conservation. 2008;23:5–17.

[53] Kappeler P, Rasoloarison R. Microcebus, mouse lemurs, Tsidy The natural history of Madagascar University of Chicago Press, Chicago 2003:1310–5.

[54] Burivalova Z, Şekercioğlu Çağan H, Koh Lian P. Thresholds of Logging Intensity to Maintain Tropical Forest Biodiversity. Current Biology. 2014;24(16):1893–8. 10.1016/j.cub.2014.06.065 25088557

[55] Laurance WF, Useche DC, Rendeiro J, Kalka M, Bradshaw CJA, Sloan SP, et al Averting biodiversity collapse in tropical forest protected areas. Nature. 2012;489(7415):290–4. 10.1038/nature11318 22832582

[56] García G, Goodman SM. Hunting of protected animals in the Parc National d'Ankarafantsika, north-western Madagascar. Oryx. 2003;37(01):115–8.

[57] Loveridge AJ, Searle AW, Murindagomo F, Macdonald DW. The impact of sport-hunting on the population dynamics of an African lion population in a protected area. Biological Conservation. 2007;134(4):548–58.

[58] Datta A, Anand MO, Naniwadekar R. Empty forests: Large carnivore and prey abundance in Namdapha National Park, north-east India. Biological Conservation. 2008;141(5):1429–35.

[59] Butler RA. Extensive logging, lemur hunting in Madagascar national park despite moratorium 16 Nov 2010. Available: http://news.mongabay.com/2010/1116-masoala_rosewood.html. Accessed 28 October 2014.

[60] Johnson S. Ambositra-Vondrozo Corridor In: Schwitzer C, Mittermeier R, Davies N, Johnson S, Ratsimbazafy J, Razafindramanana J, et al, editors. Lemurs of Madagascar: A Strategy for Their Conservation 2013–2016. Bristol, UK:: IUCN SSC Primate Specialist Group, Bristol Conservation and Science Foundation, and Conservation International; 2013 p. 86–9.

[61] Amarasekare P, Hoopes MF, Mouquet N, Holyoak M. Mechanisms of coexistence in competitive metacommunities. American Naturalist. 2004;164(3):310–26. 15478087 10.1086/422858

[62] Hudgens BR, Haddad NM. Predicting Which Species Will Benefit from Corridors in Fragmented Landscapes from Population Growth Models. The American Naturalist. 2003;161(5):808–20. 12858286 10.1086/374343

[63] Louis EE, Ratsimbazafy JH, Razakamaharauo VR, Pierson DJ, Barber RC, Brenneman RA. Conservation genetics of black and white ruffed lemurs, Varecia variegata, from Southeastern Madagascar. Animal Conservation. 2005;8(1):105–11.

[64] Baden AL, Holmes SM, Johnson SE, Engberg SE, Louis EE, Bradley BJ. Species-level view of population structure and gene flow for a critically endangered primate (Varecia variegata). Ecology and Evolution. 2014;4(13):2675–92. 10.1002/ece3.1119 25077019 PMC4113292

[65] Holmes S, Baden A, Brenneman R, Engberg S, Louis E Jr, Johnson S. Patch size and isolation influence genetic patterns in black-and-white ruffed lemur (Varecia variegata) populations. Conservation Genetics. 2013;14(3):615–24.

[66] Haddad NM, Brudvig LA, Damschen EI, Evans DM, Johnson BL, Levey DJ, et al Potential negative ecological effects of corridors. Conservation biology: the journal of the Society for Conservation Biology. 2014;28(5). 10.1111/cobi.12323 25115896

