# Supplementary material for: Large-Scale Habitat Corridors for Biodiversity Conservation: A Forest Corridor in Madagascar
Source: PLoS One. 2015 Jul 22;10(7):e0132126. doi: 10.1371/journal.pone.0132126 (PMC4511669; doi:10.1371/journal.pone.0132126)
Supplement: S1 Fig — (PDF) [file pone.0132126.s001.pdf]

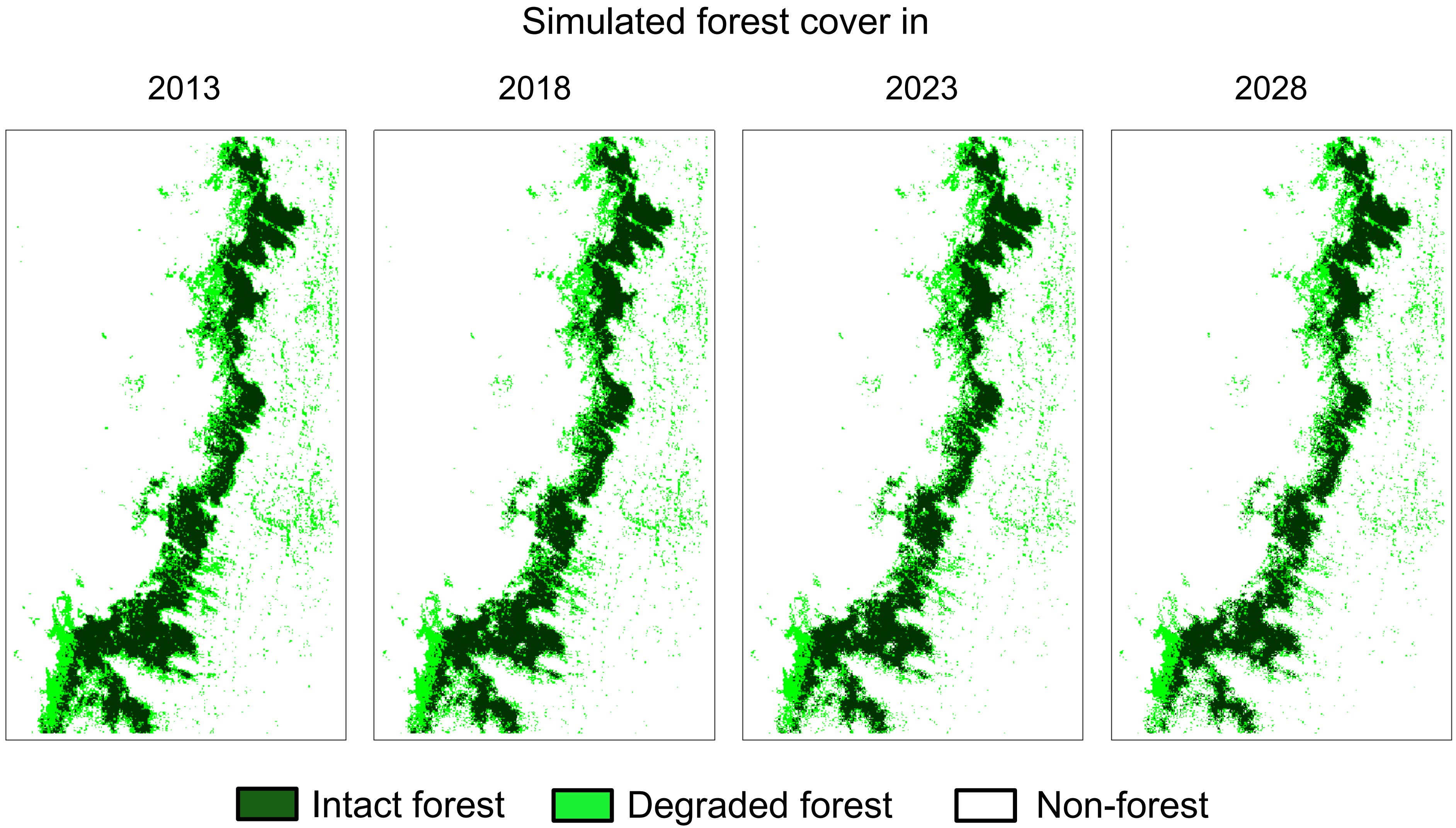
Simulated forest cover in
2013
2018
2023
2028
Intact forest
Degraded forest
Non-forest

Figure A

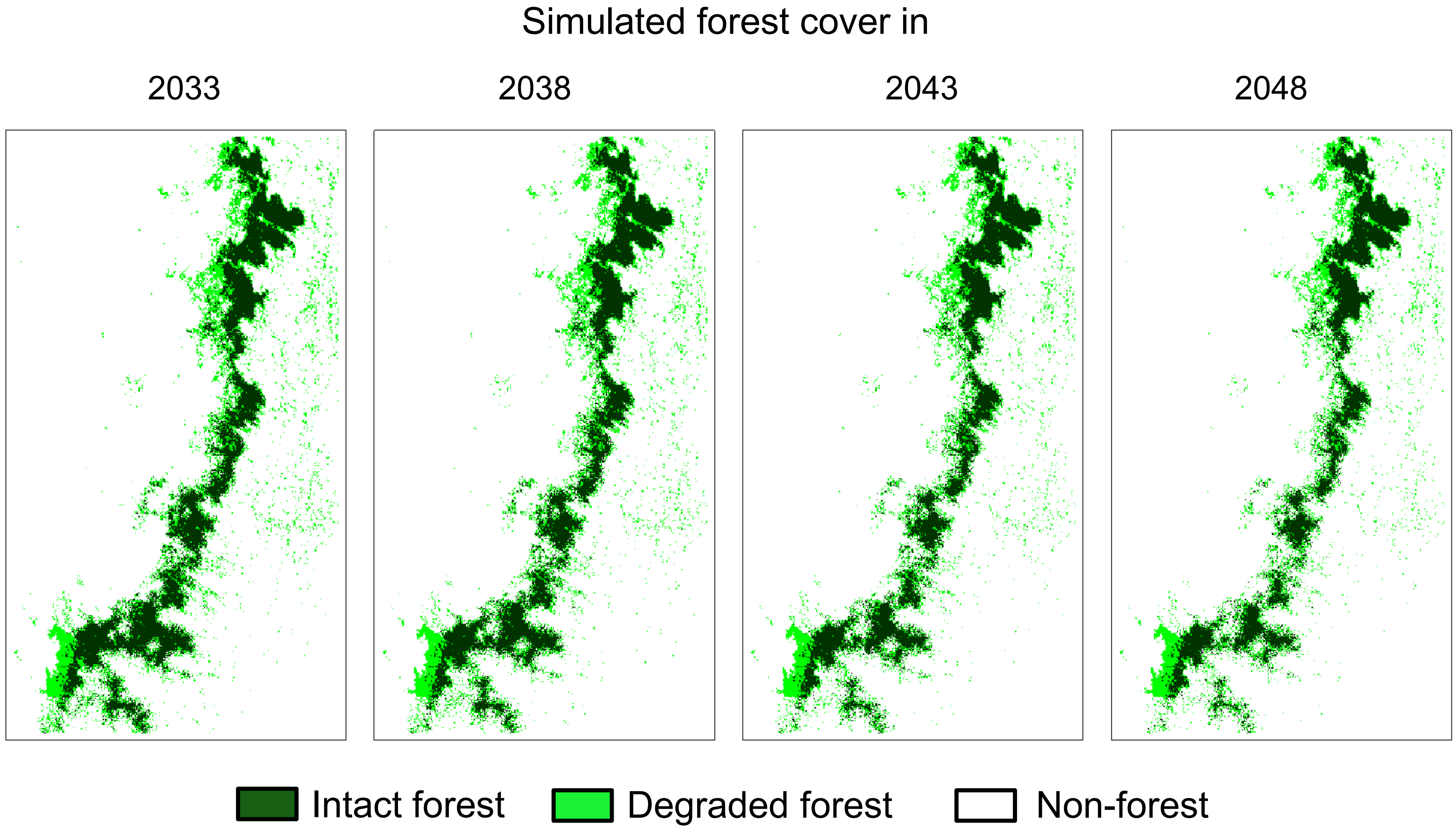
Simulated forest cover in
2033
2038
2043
2048
Intact forest
Degraded forest
Non-forest

Figure B

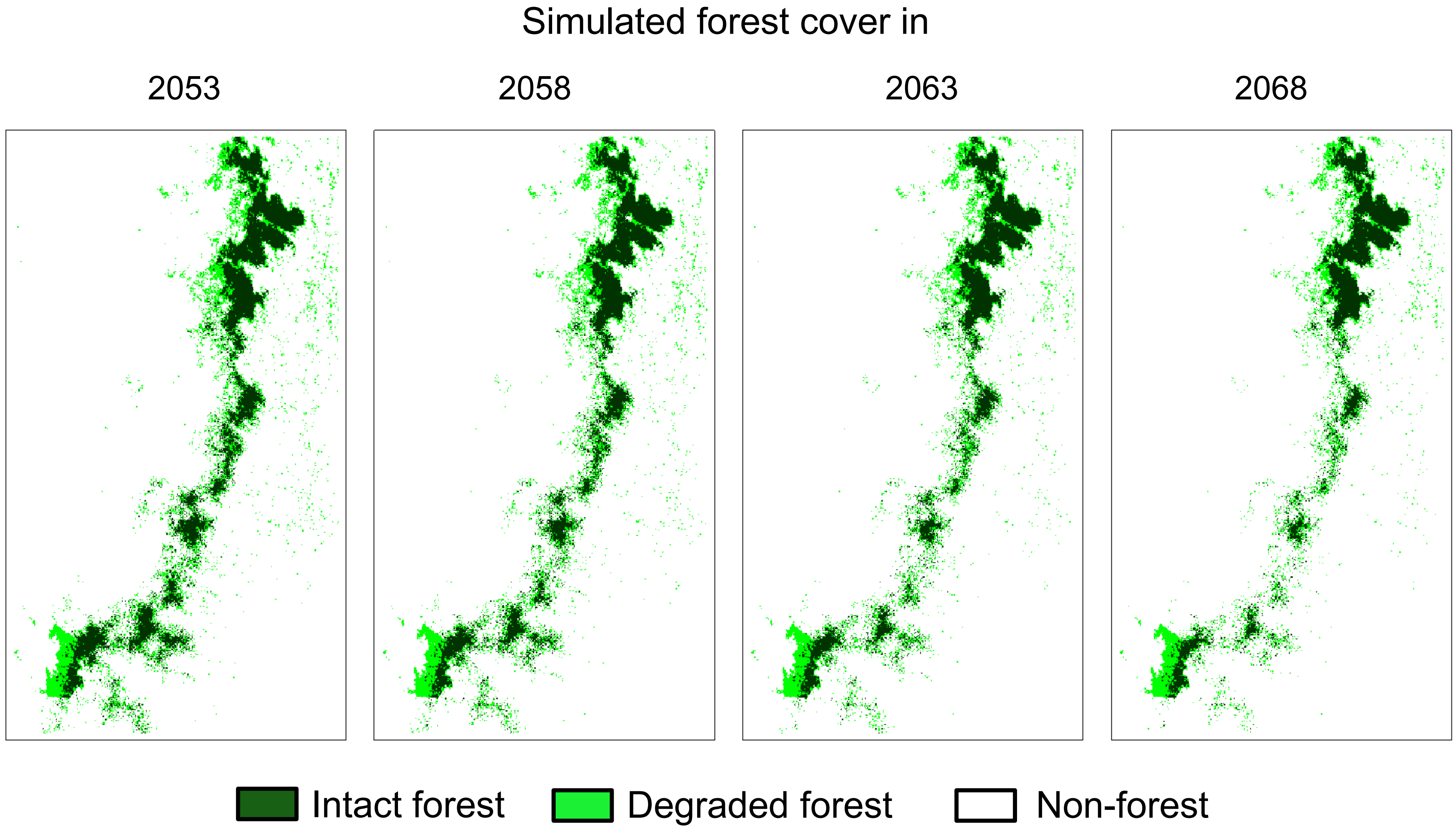

Simulated forest cover in
2053
2058
2063
2068
Intact forest
Degraded forest
Non-forest

Figure C

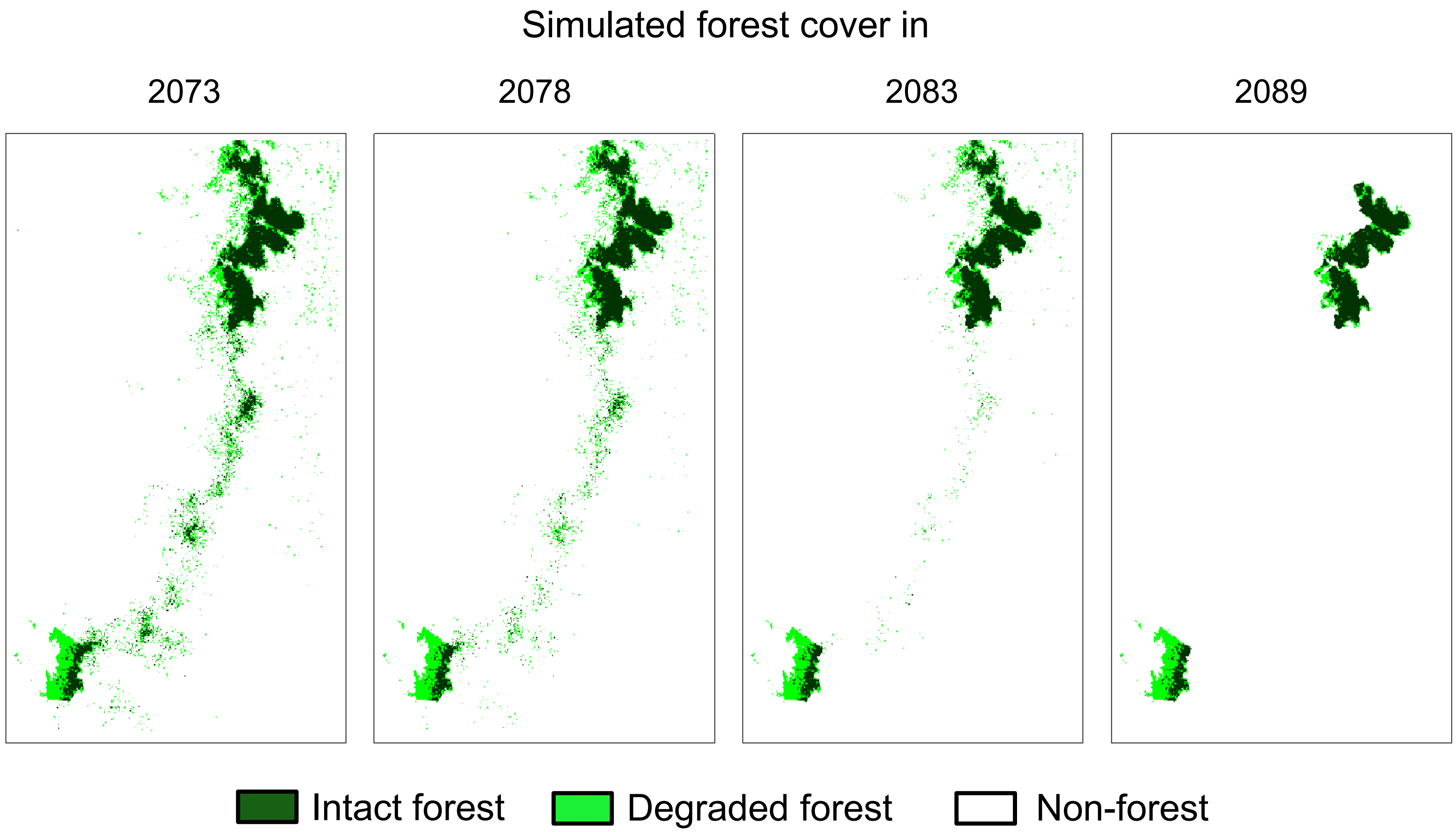
Simulated forest cover in
2073
2078
2083
2089
Intact forest
Degraded forest
Non-forest

Figure D
