# Supplementary figures and images for: Large-Scale Habitat Corridors for Biodiversity Conservation: A Forest Corridor in Madagascar
Source: PLoS One. 2015 Jul 22;10(7):e0132126. doi: 10.1371/journal.pone.0132126 (PMC4511669; doi:10.1371/journal.pone.0132126)

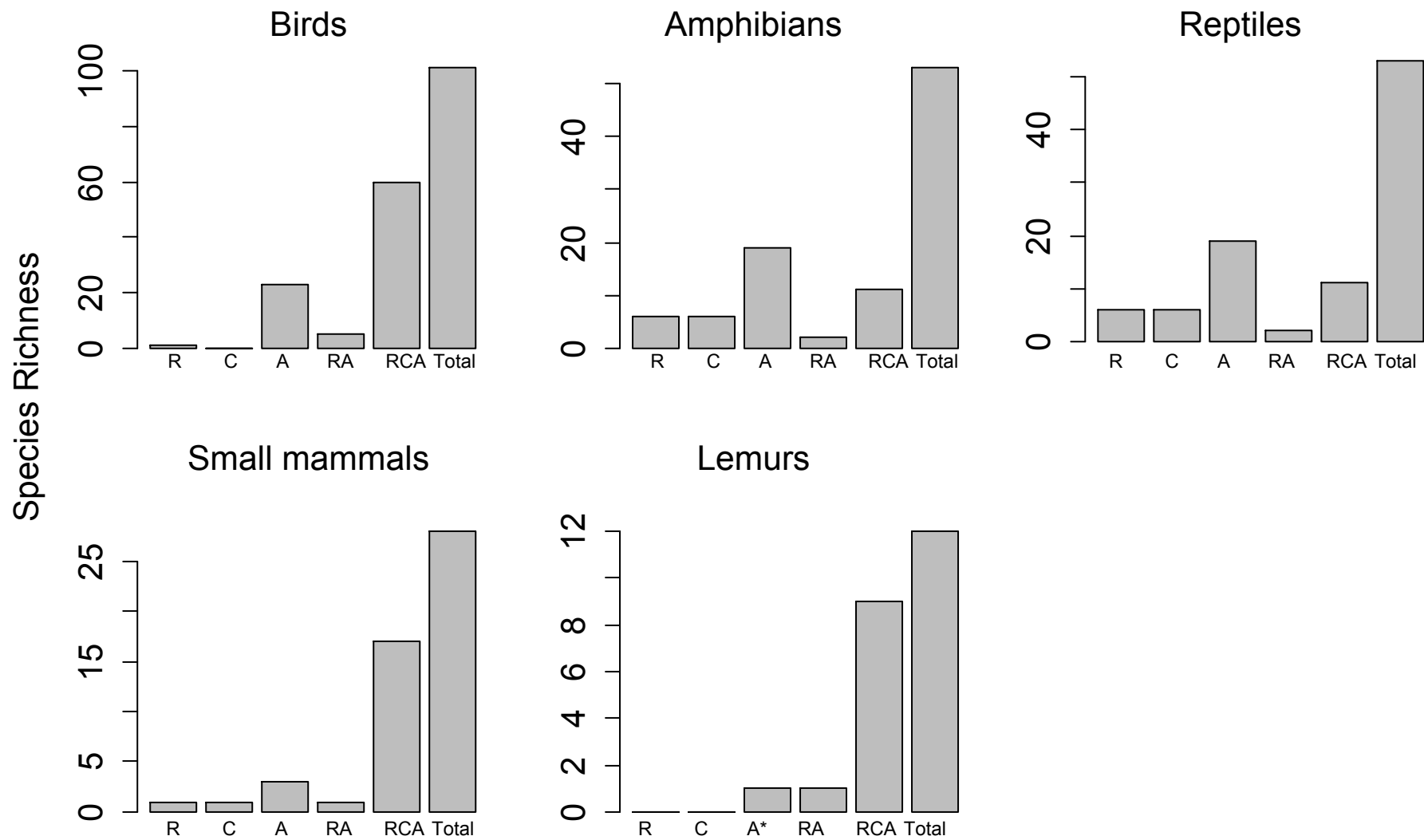

Figure A

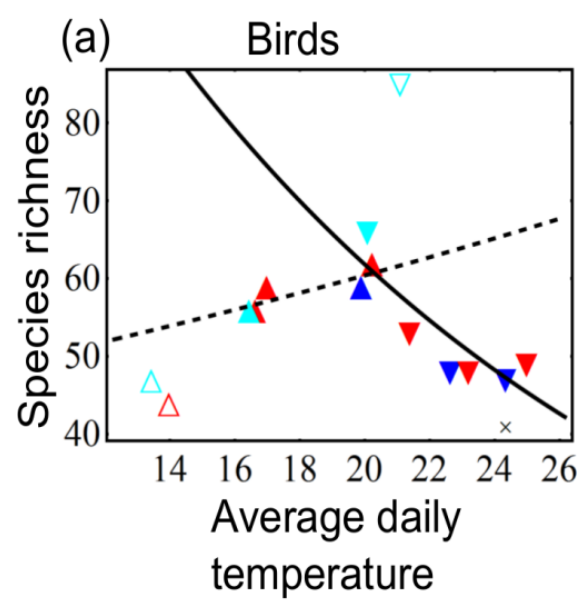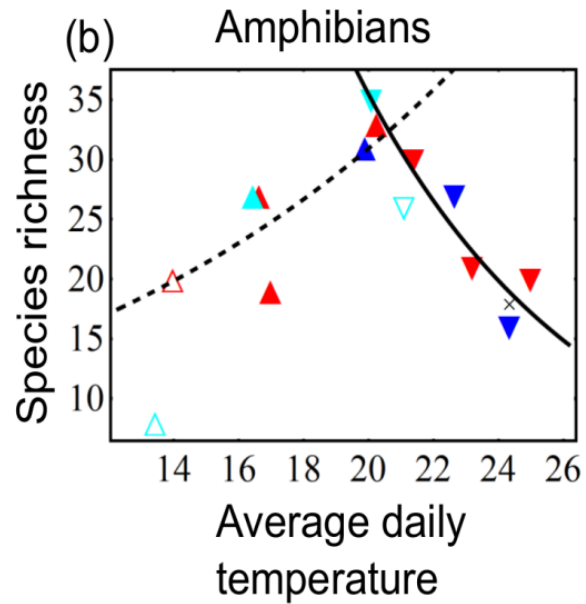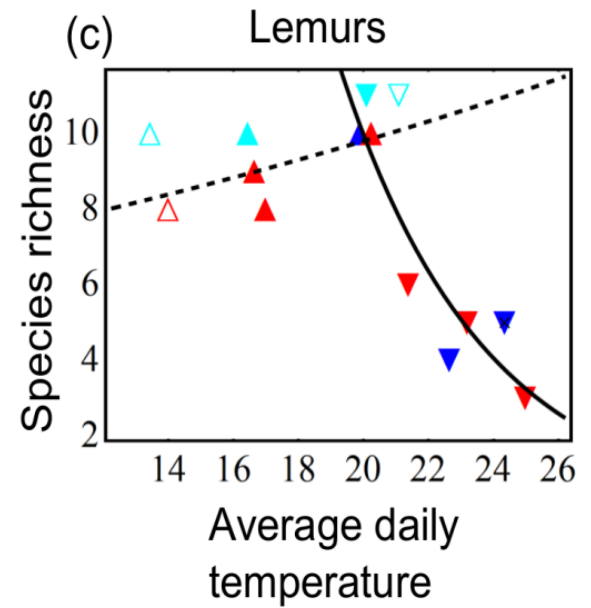

Figure B

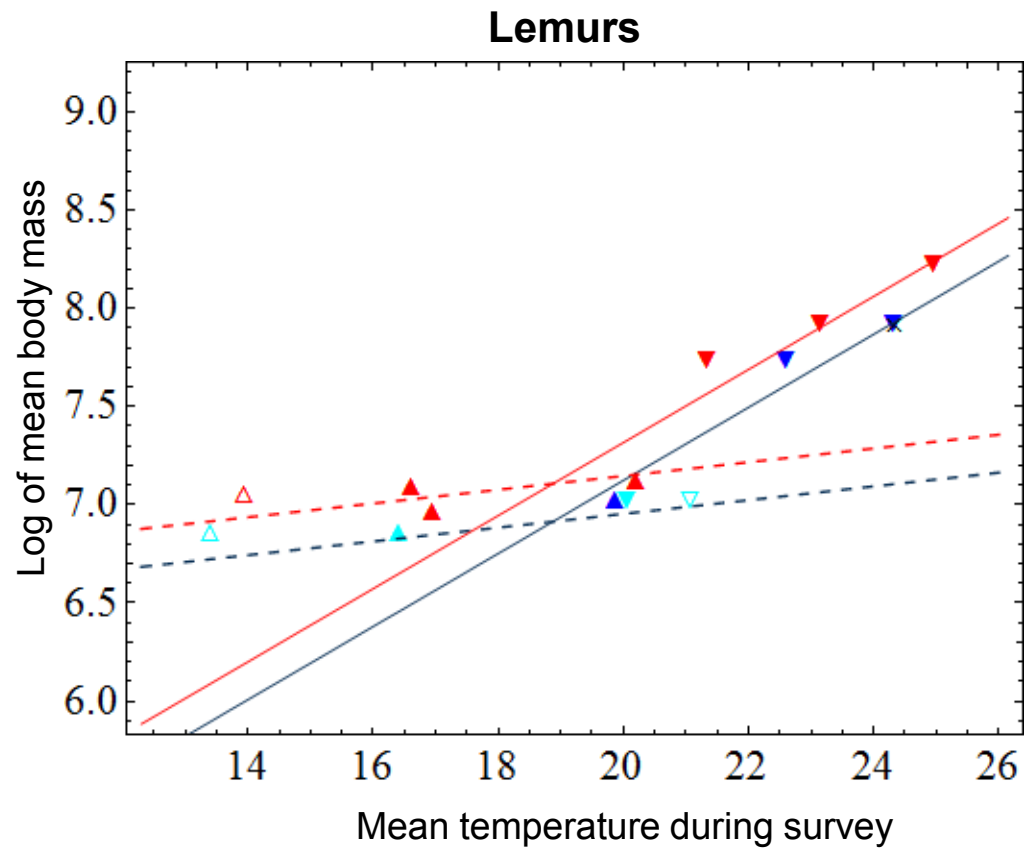

Figure C

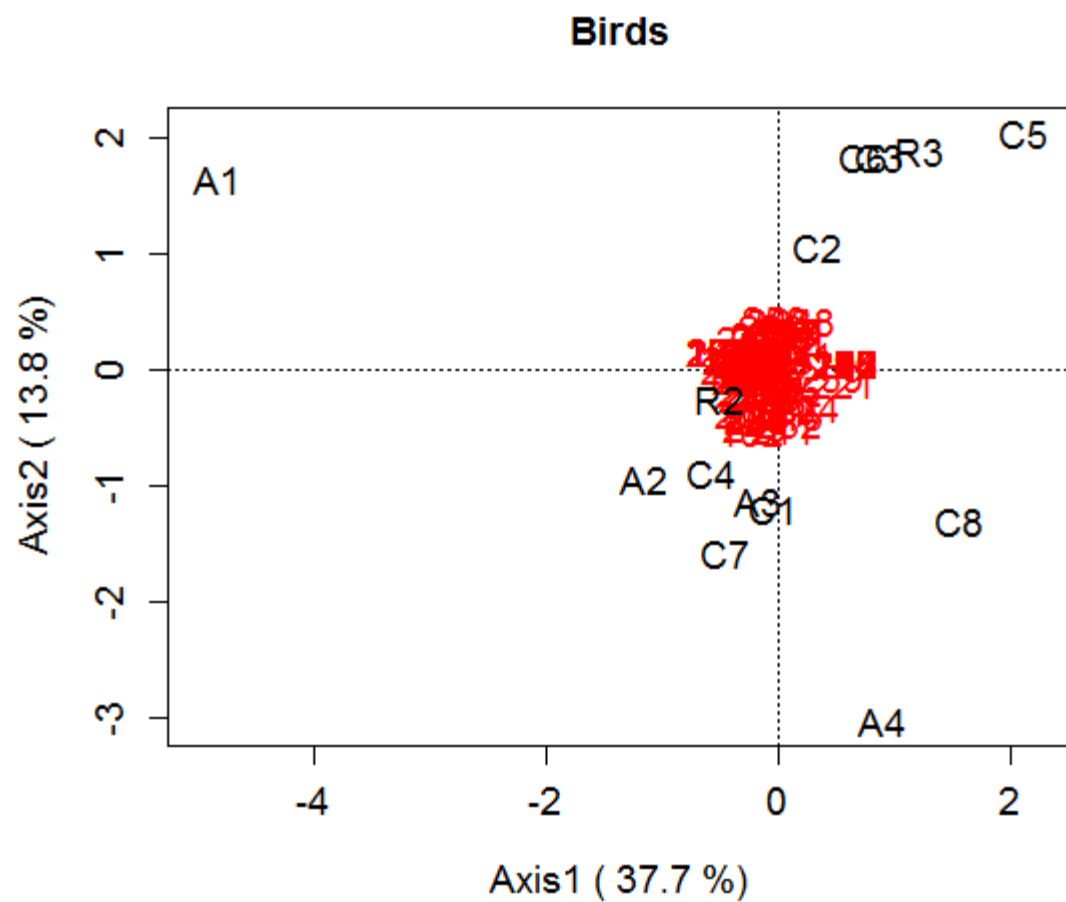

Figure D

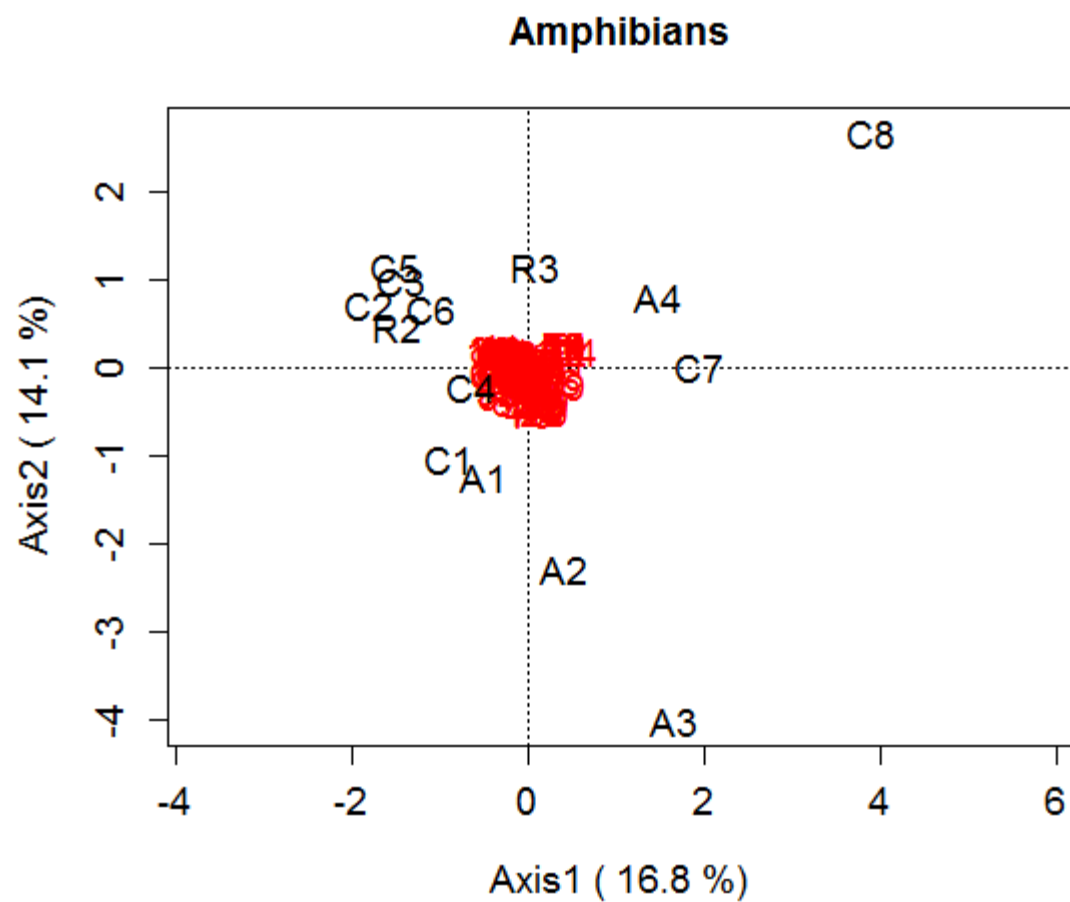

Figure E

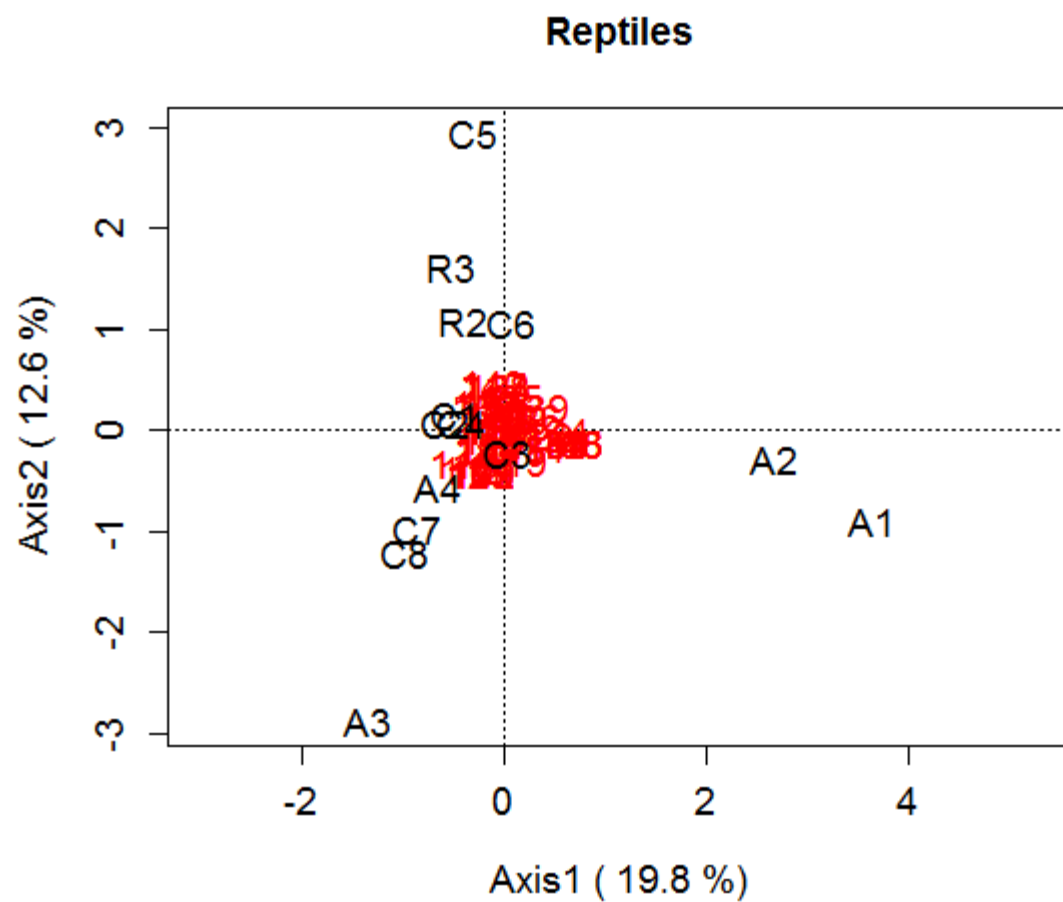

Figure F

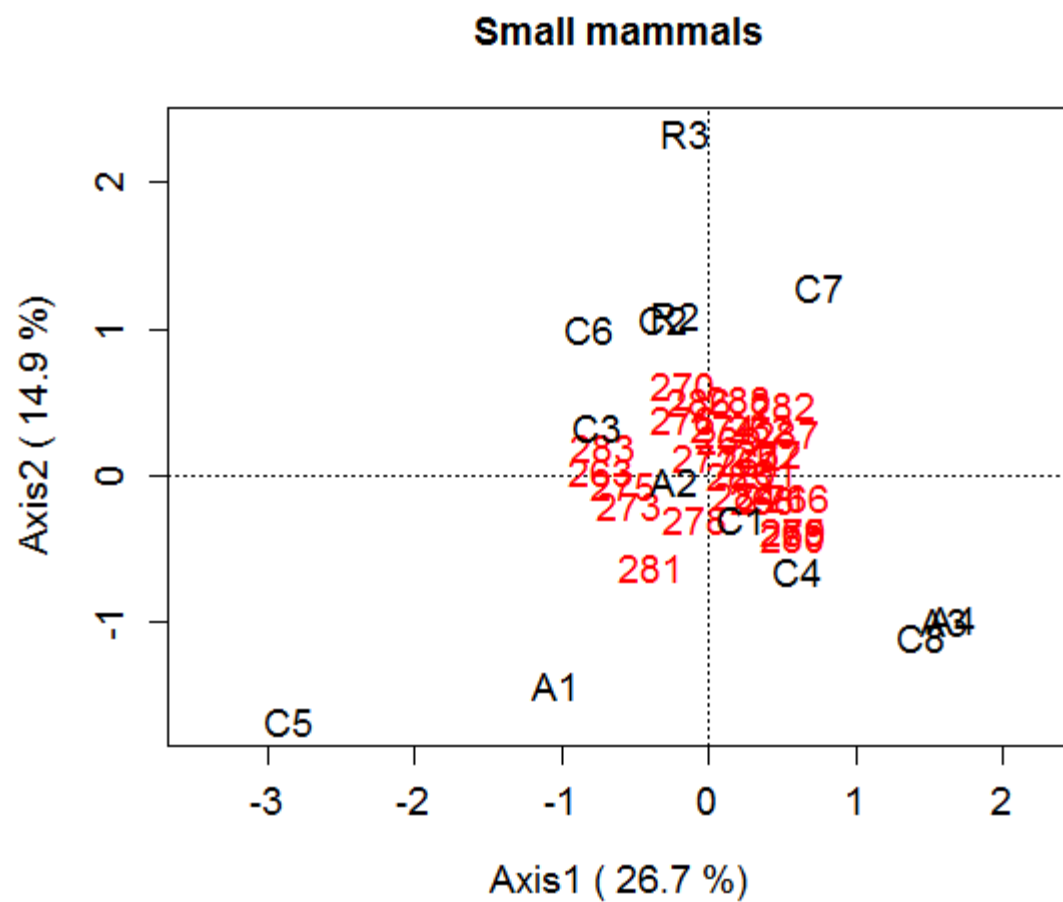

Figure G

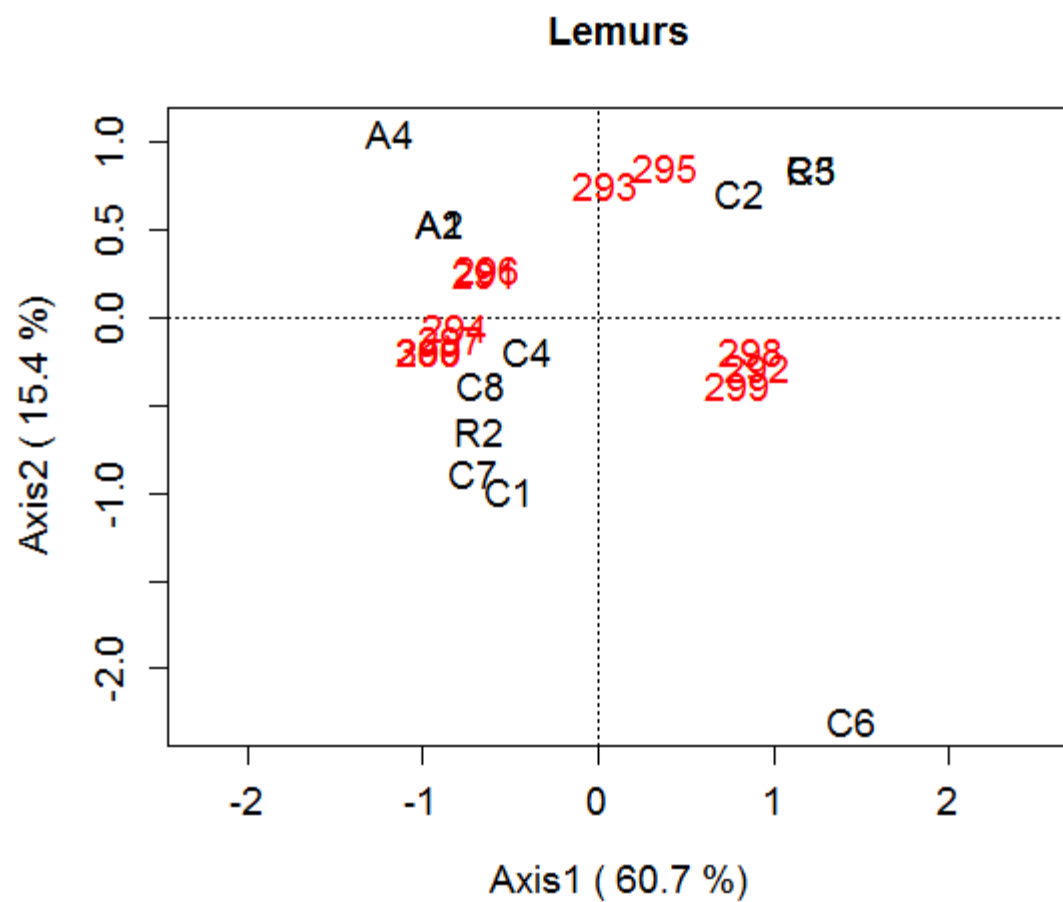

Figure H

Supplement: S2 Fig — Fig A in S2 Fig shows the number of species unique to Ranomafana NP (R), Corridor (C), and Andringitra NP (A). RA denotes the number of common species to Ranomafana NP and Andringitra NP. RCA denotes the number of species present across the entire landscape. Fig B in S2 Fig shows the Poisson regression of species richness per site as a function of average temperature during the survey. Dashed (solid) lines with up-ward (down-ward) pointing triangles show the data for sites above (below) 1000 m asl. Blue, red and cyan denote sites in the Ranomafana park, the corridor and the Andringitra park, respectively. Empty symbol indicates missing temperature data that were extrapolated using the nearest site for visualization. Model comparisons and P values are in S3 File. Fig C in S2 Fig shows the effect of temperature (mT), altitude above and below 1000m asl (altC), and whether the site belongs to the corridor or not (PC) on the logarithm of body size of lemurs using Poisson regression. Red (indigo) fitted lines are for sites in corridor (park). Solid (dashed) lines are for sites below (above) 1000 m. The symbols are as in Fig B in S2 Fig. Figs D-H in S2 Fig show the results of the principal component analysis for each taxonomic group. (PDF) [file pone.0132126.s002.pdf]
