# Supplementary material for: Large-Scale Habitat Corridors for Biodiversity Conservation: A Forest Corridor in Madagascar
Source: PLoS One. 2015 Jul 22;10(7):e0132126. doi: 10.1371/journal.pone.0132126 (PMC4511669; doi:10.1371/journal.pone.0132126)
Supplement: S3 Fig — shows equilibrium occupancy for both Ranomafana NP and Andringitra NP for intact forest specialists (blue), degraded forest specialists (red), and generalists (black). Solid, dashed and dotted lines represent, passive dispersal, active without gap-avoidance and with gap-avoidance, respectively. Error bars show minimum and maximum value for different replicates. In Figs A-B in S3 Fig, c = 0.2, e = 0.1, μ = 0, σ 2 = 1. Figs C-D in S3 Fig show the outcome of the simulation for larger values of colonization and extinction rates without regional stochasticity. (PDF) [file pone.0132126.s003.pdf]

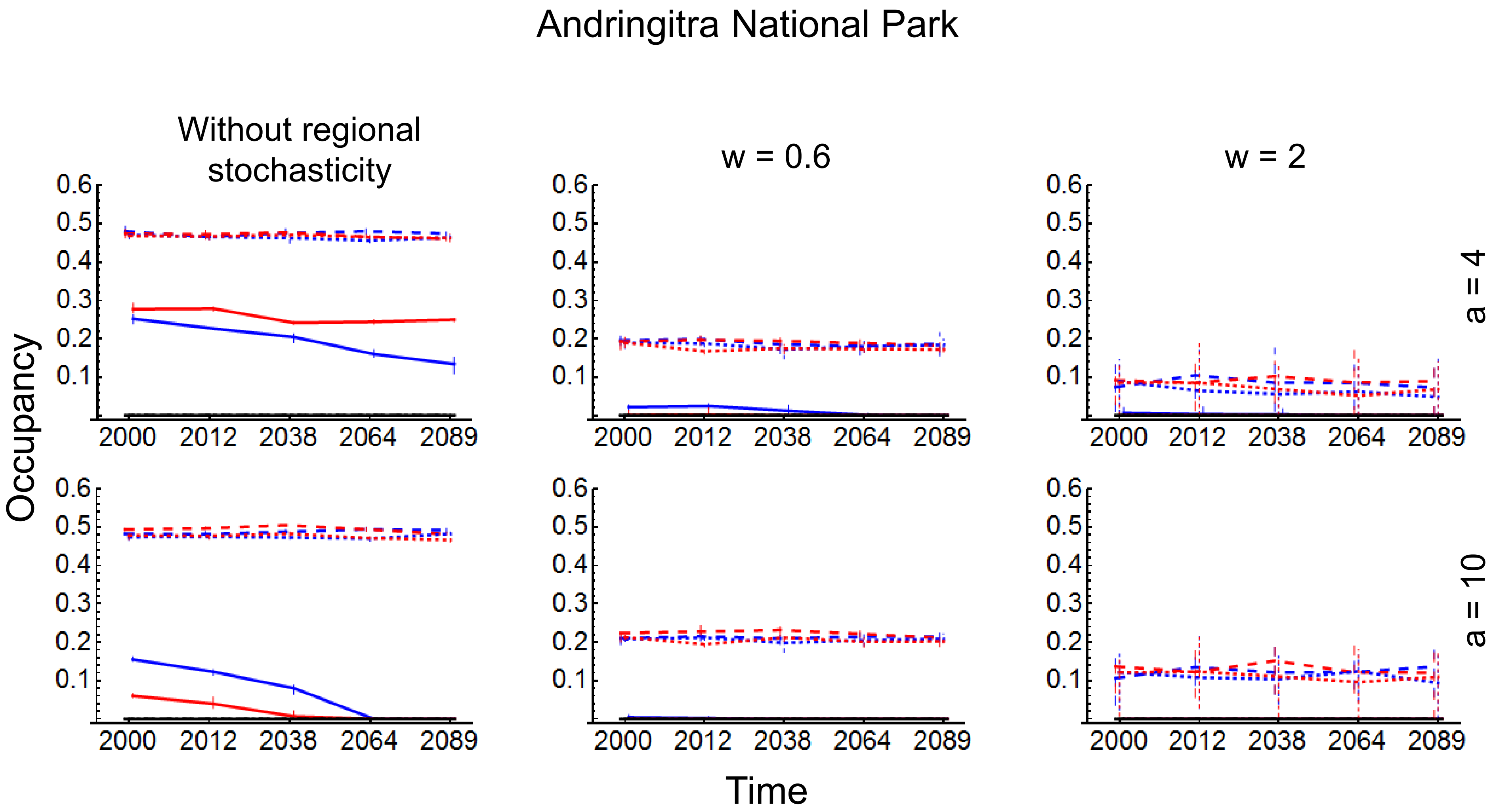
Andringitra National Park
Without regional stochasticity
w = 0.6
w = 2
a = 4
a = 10
Occupancy
Time
0.6
0.5
0.4
0.3
0.2
0.1
2000
2012
2038
2064
2089

Figure A

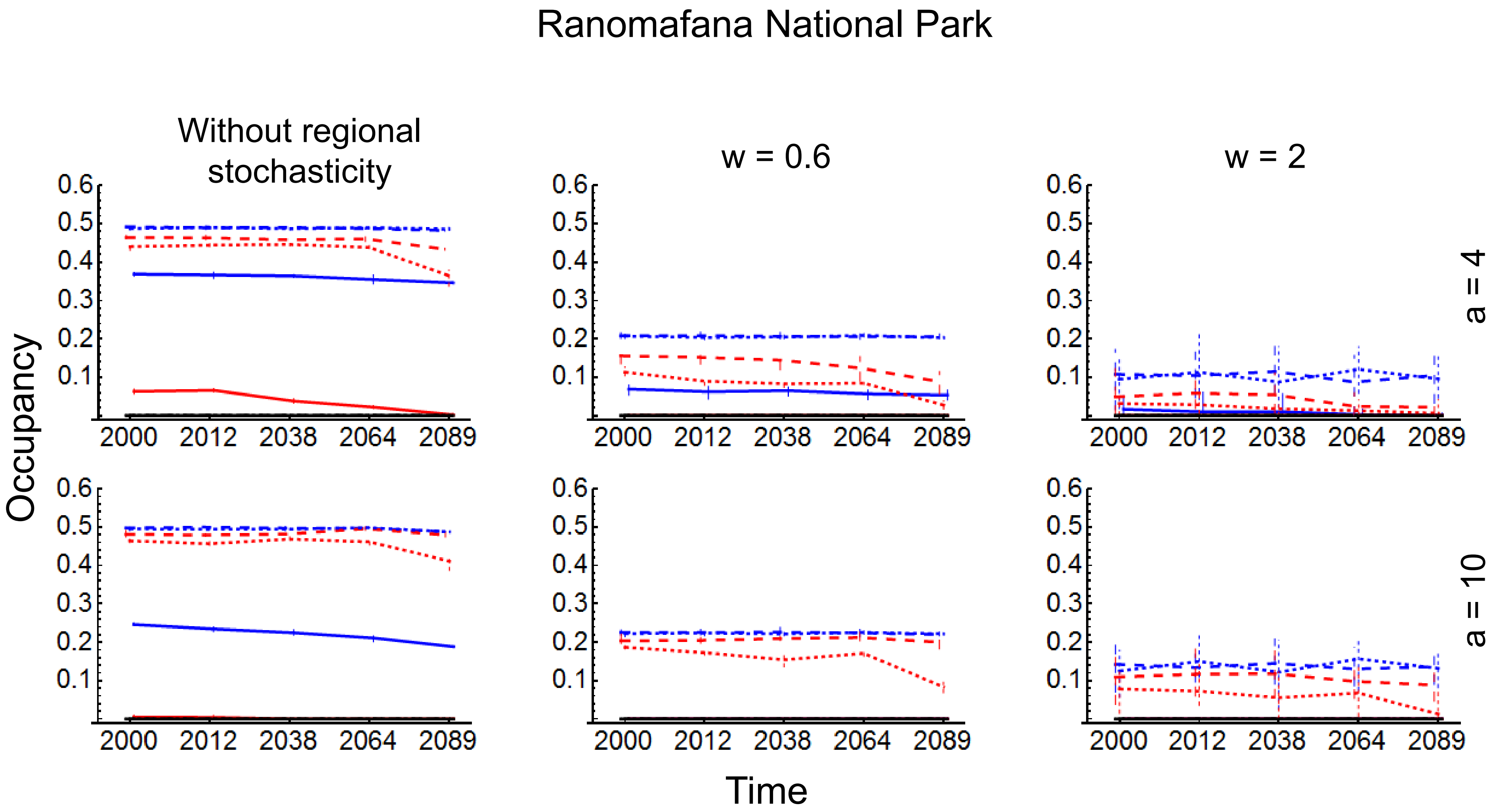

Ranomafana National Park
Without regional stochasticity
w = 0.6
w = 2
a = 4
a = 10
Occupancy
Time
0.6
0.5
0.4
0.3
0.2
0.1
2000
2012
2038
2064
2089


Figure B

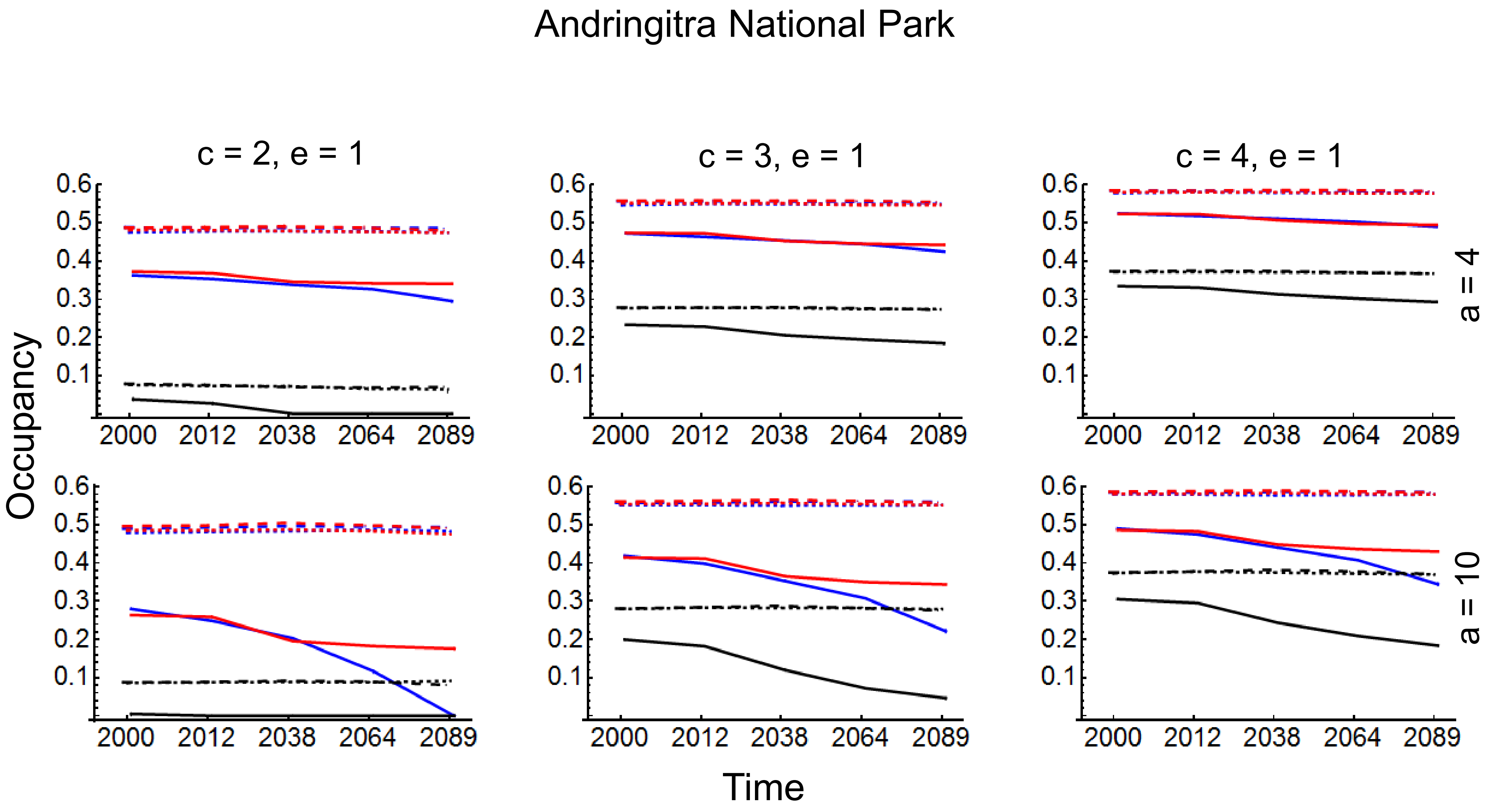

Andringitra National Park
c = 2, e = 1
c = 3, e = 1
c = 4, e = 1
0.6
0.5
0.4
0.3
0.2
0.1
2000
2012
2038
2064
2089
Occupancy
Time
a = 4
a = 10


Figure C

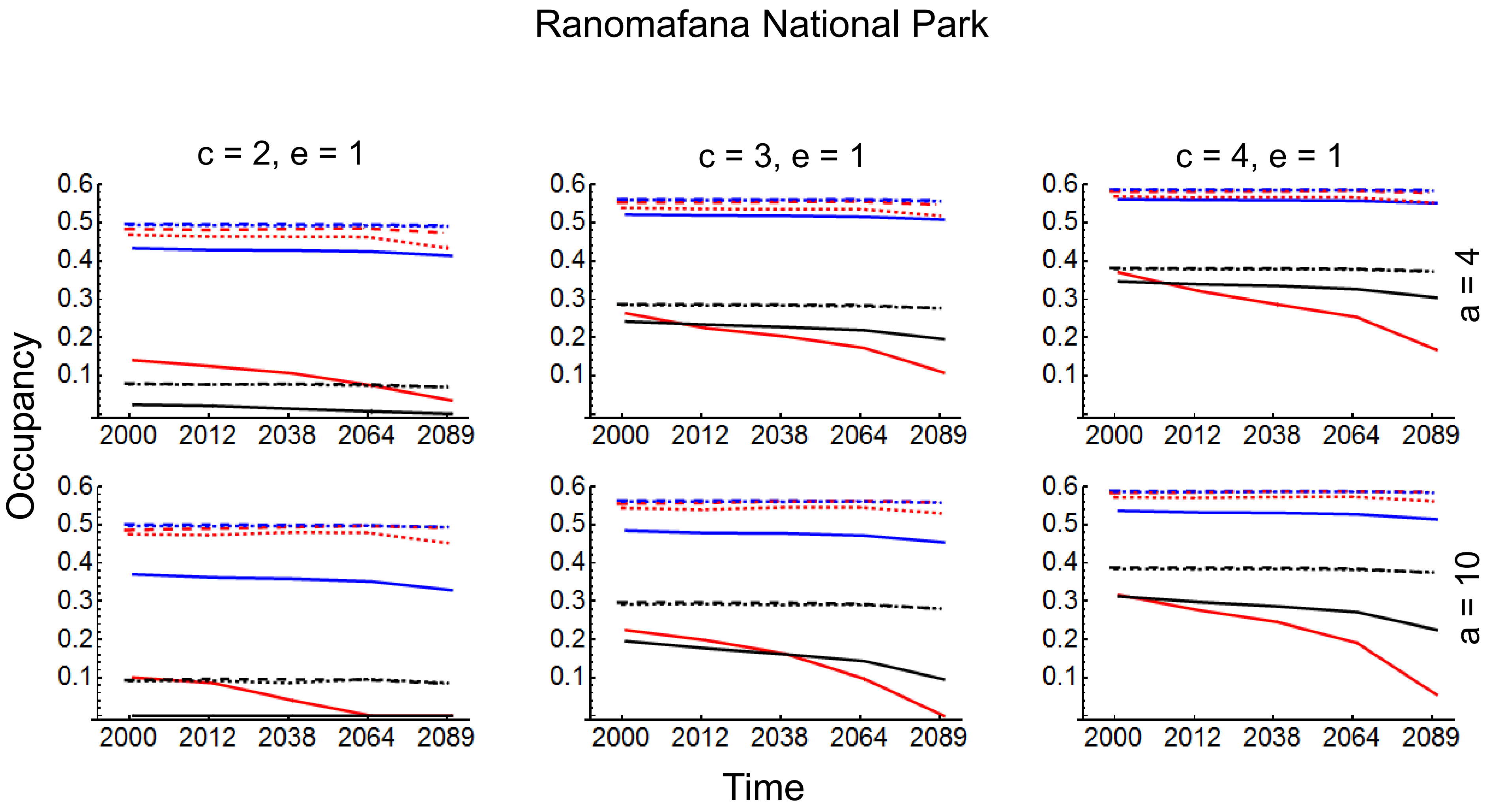

Ranomafana National Park
c = 2, e = 1
c = 3, e = 1
c = 4, e = 1
Occupancy
0.6
0.5
0.4
0.3
0.2
0.1
2000
2012
2038
2064
2089
a = 4
a = 10
Time


Figure D
