# Supplementary material for: Large-Scale Habitat Corridors for Biodiversity Conservation: A Forest Corridor in Madagascar
Source: PLoS One. 2015 Jul 22;10(7):e0132126. doi: 10.1371/journal.pone.0132126 (PMC4511669; doi:10.1371/journal.pone.0132126)
Supplement: S4 Fig — The full result for Fig 6 is shown in Fig A in S4 Fig, which includes degraded forest specialists and forest generalists. Figs B-E in S4 Fig show the influence of high values of colonization and extinction rates, and dispersal range on transient time without regional stochasticity setting initial occupancy in Ranomafana NP or Andringitra NP. Intact forest specialists, degraded forest specialists, and generalists are represented by blue, red, and black, respectively. Solid, dashed and dotted lines represent, passive dispersal, active without gap-avoidance and with gap-avoidance, respectively. Lines on the ≥2000-mark mean that the other park was never reached after 2000 time steps. Solid dots, empty dots, and empty squares above Ext (upper gray line) mean that the passive, active without gap-avoidance, and active with gap-avoidance dispersers went extinct. The figures show that higher values of colonization and extinction rates reduce the difference among species and among modes of dispersal. Fig E in S4 Fig shows that transient time becomes independent of corridor quality for extremely large mean dispersal. Fig F in S4 Fig shows the influence of the correlation in the spatial scale in environmental stochasticity (w) on transient time. Black represents models without regional stochasticity. Green, red, and blue represent model with regional stochasticity with w = 0.1, 1, and 2, respectively. Fig F in S4 Fig shows that including regional stochasticity leads to higher transient time. Small to moderate value of w yield similar transient times; very large w however lengthens transient time. (PDF) [file pone.0132126.s004.pdf]

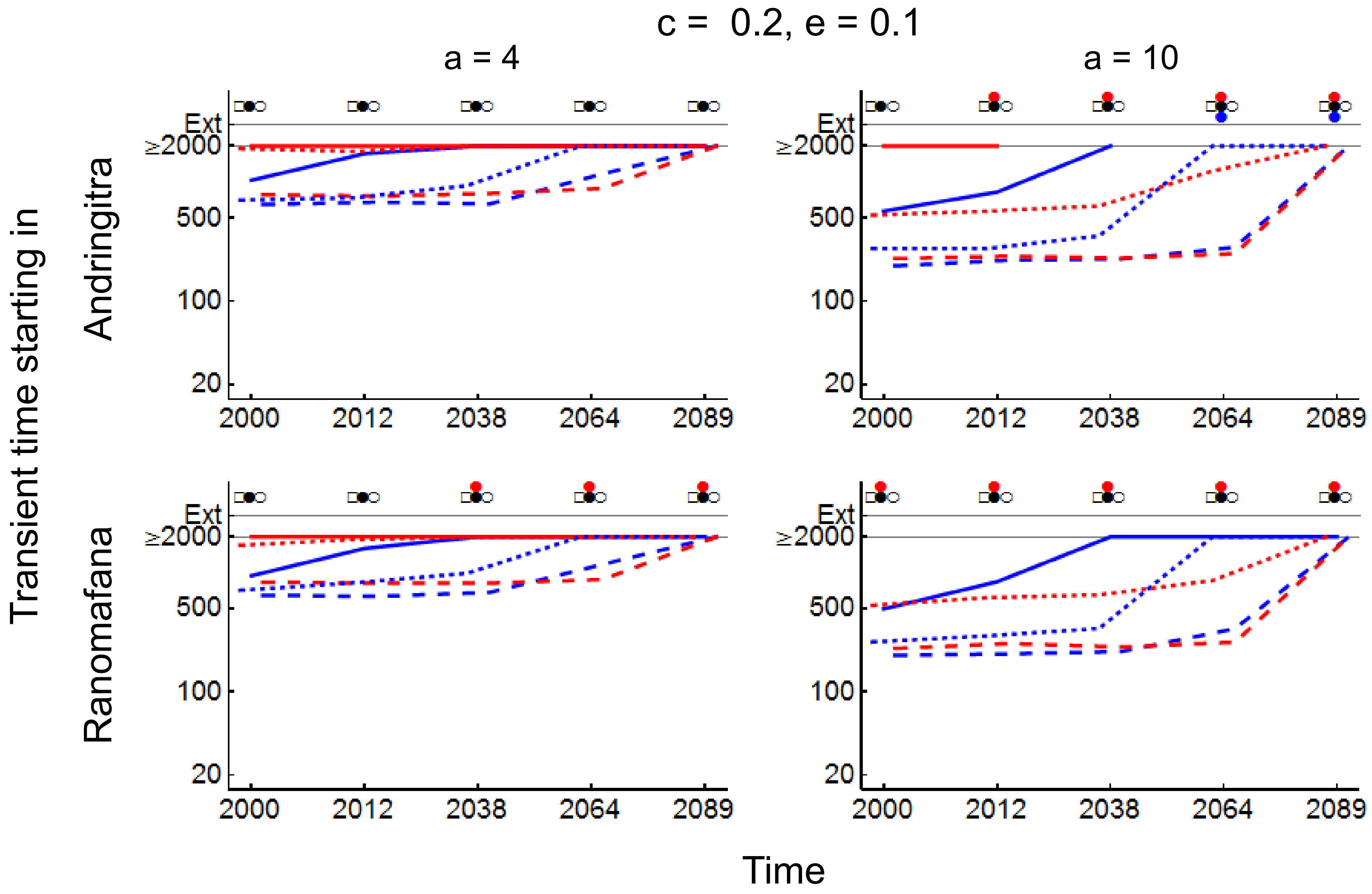
c = 0.2, e = 0.1
a = 4
a = 10
Transient time starting in
Andringitra
Ranomafana
Ext
≥2000
500
100
20
2000
2012
2038
2064
2089
Time

Figure A

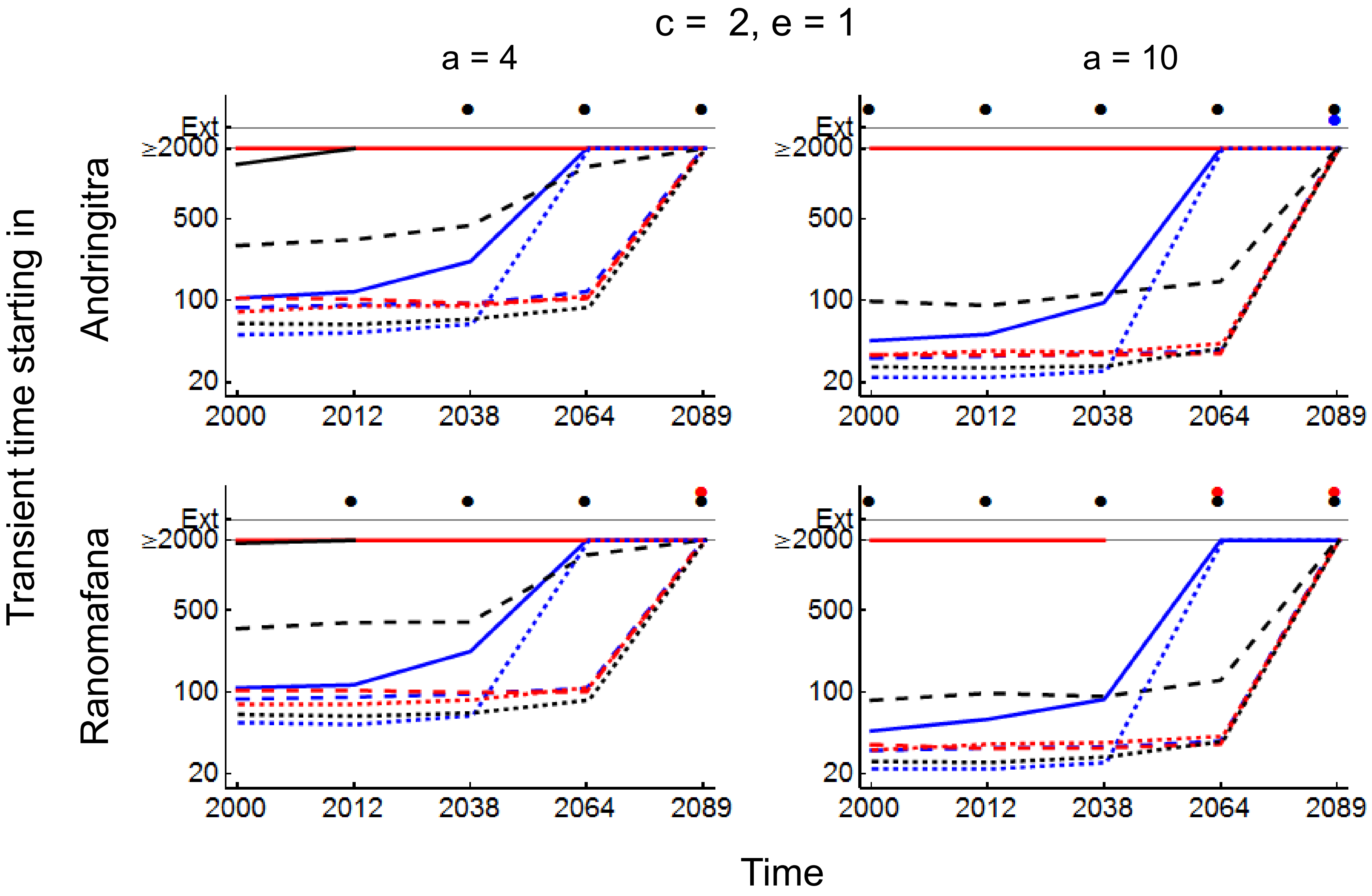

c = 2, e = 1
a = 4
a = 10
Transient time starting in
Andringitra
Ranomafana
Ext
≥2000
500
100
20
2000
2012
2038
2064
2089
Time

Figure B

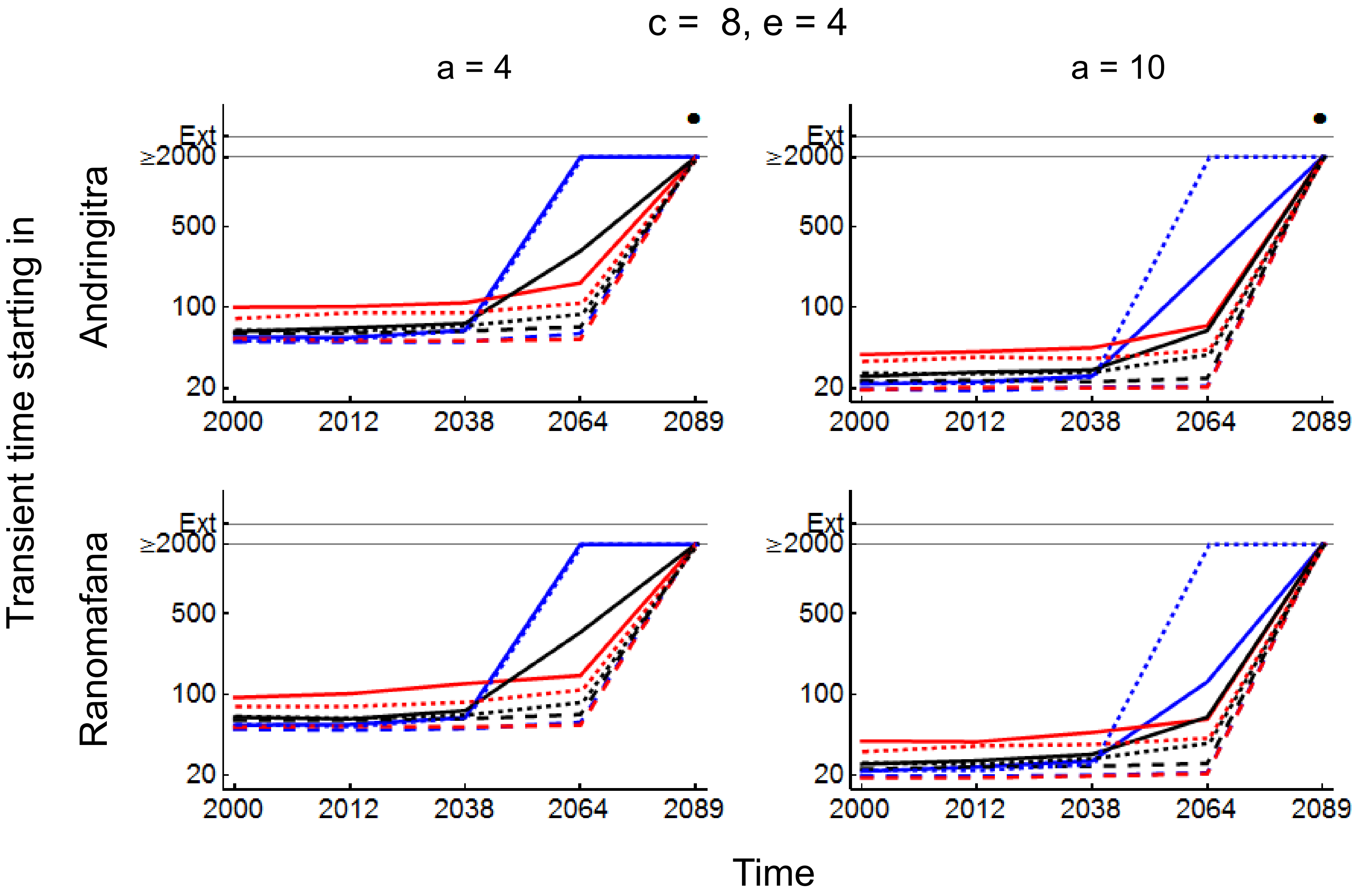

c = 8, e = 4
a = 4
a = 10
Transient time starting in
Andringitra
Ranomafana
Ext
≥2000
500
100
20
2000
2012
2038
2064
2089
Time

Figure C

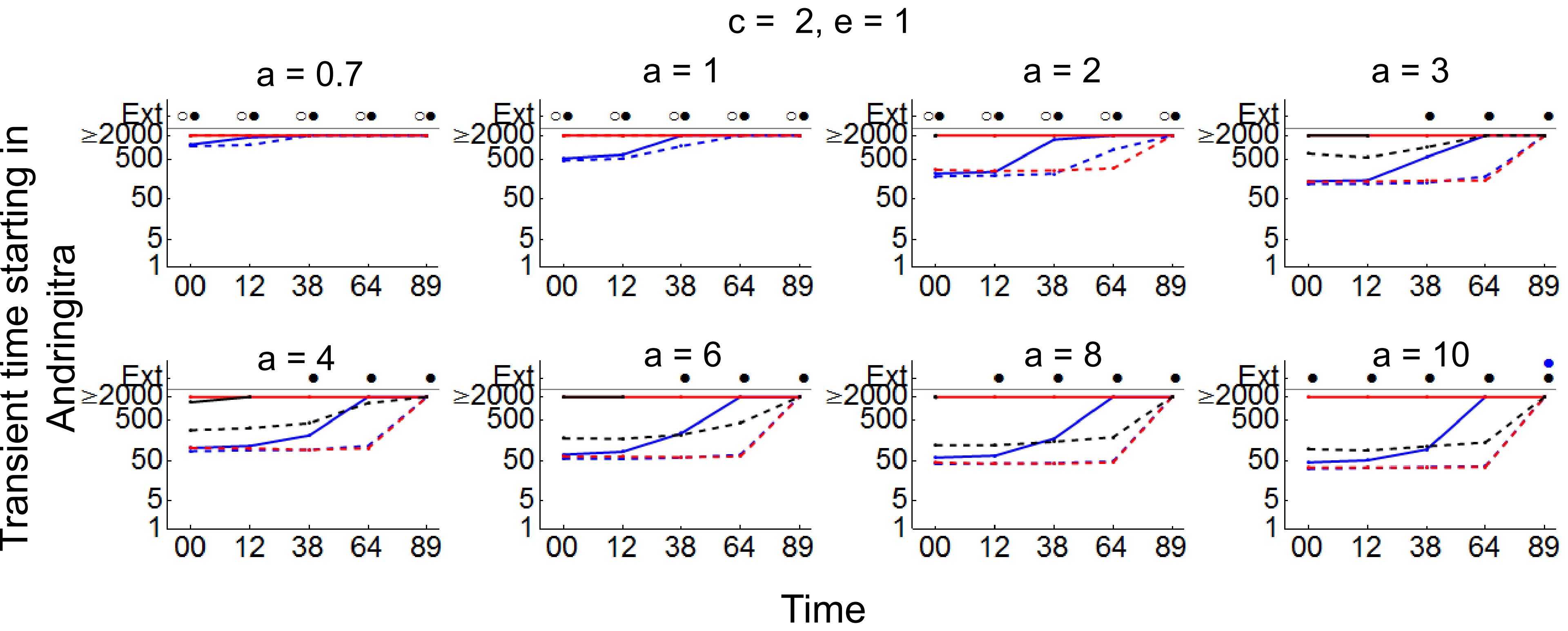

c = 2, e = 1
a = 0.7
a = 1
a = 2
a = 3
a = 4
a = 6
a = 8
a = 10
Ext
≥2000
500
50
5
1
00
12
38
64
89
Transient time starting in Andringitra
Time

Figure D

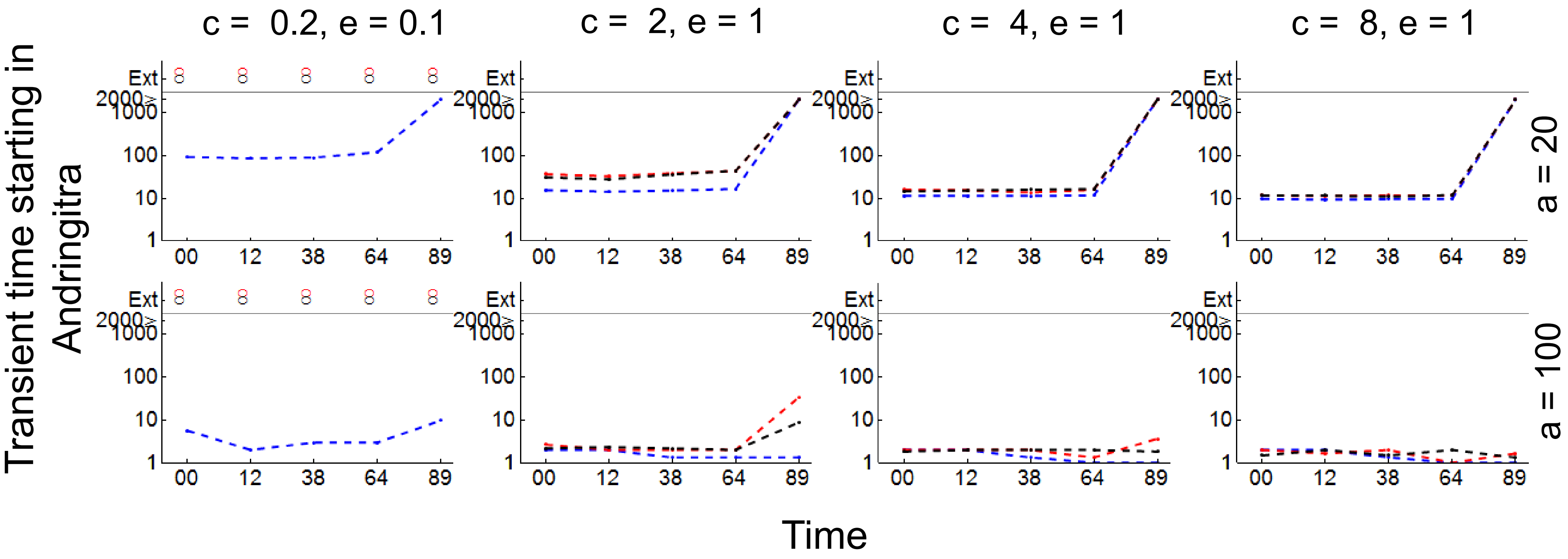
c = 0.2, e = 0.1
c = 2, e = 1
c = 4, e = 1
c = 8, e = 1
Transient time starting in Andringitra
Ext
2000≥
1000
100
10
1
00
12
38
64
89
a = 20
a = 100
Time

Figure E

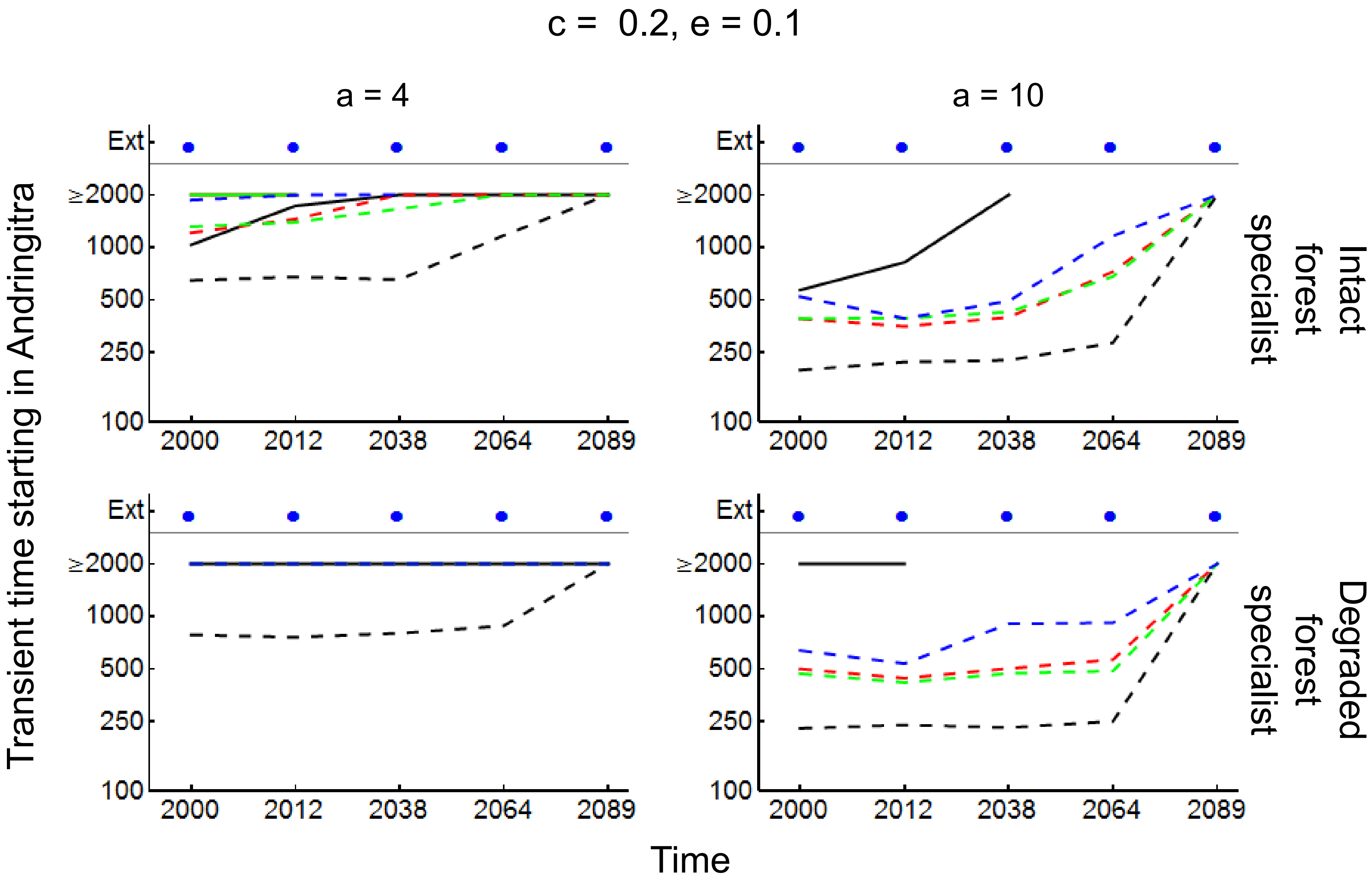

c = 0.2, e = 0.1
a = 4
a = 10
Ext
≥2000
1000
500
250
100
2000
2012
2038
2064
2089
Intact forest specialist
Degraded forest specialist
Transient time starting in Andringitra
Time

Figure F
