# Supplementary material for: Large-Scale Habitat Corridors for Biodiversity Conservation: A Forest Corridor in Madagascar
Source: PLoS One. 2015 Jul 22;10(7):e0132126. doi: 10.1371/journal.pone.0132126 (PMC4511669; doi:10.1371/journal.pone.0132126)
Supplement: S1 File — (DOCX) [file pone.0132126.s005.docx]

**Detailed description of the deforestation algorithm.**

We generate a deforestation process based on the reclassified map of 2012 (see the main text). To do so, we assume that deforestation rate of a cell *i* depends on the altitude and the habitat quality in the neighborhood, and is given by

$$r(c_{i})=p_{i}\sum_{c_{j}\in nb(c_{i})} d(c_{i},c_{j})q(c_{j}).$$

Here *nb*(*c_i_*) is the neighborhood of cell *i* and consists of its first and second rings (8 and 16 cells respectively). *d*(*c_i_*, *c_j_* ) is the inverse of the Euclidian distance between cells *i* and *j*, *q*(*c_j_*) is an indicator variable and has the value 1 if cell *j* is non-forest and 0 otherwise. *p_i_* denotes the probability of the cell *i* being destroyed based on its altitude and is obtained by computing the fraction of destroyed cells at a given altitude. For simplicity, we group altitudes into 100 m intervals.

For each round of deforestation we randomly sample *n* cells based on the rate calculated above. 6914 cells have been altered between 2000 and 2012, which on average gives 576 cells destroyed per year. If a selected cell is a degraded forest it will be turned into a non-forest cell, while if the selected cell is an intact forest cell, it may turn into degraded forest or non-forest cell. We calculated the probability of intact forest cell turning into degraded forest using the empirical results for 2000 and 2012. This probability was 0.94. The intact forest cell was transformed into non-forest with probability 0.06.
