# Supplementary material for: Large-Scale Habitat Corridors for Biodiversity Conservation: A Forest Corridor in Madagascar
Source: PLoS One. 2015 Jul 22;10(7):e0132126. doi: 10.1371/journal.pone.0132126 (PMC4511669; doi:10.1371/journal.pone.0132126)
Supplement: S2 File — (PDF) [file pone.0132126.s006.pdf]

Environmental stochasticity in the simulation model

We briefly describe some details regarding the generation of spatially autocorrelated environmental stochasticity (called regional stochasticity) in the simulation model; this approach was described in Gu et al. (2002) and Rybicki and Hanski (2013).

Computing regional stochasticity. We introduce environmental stochasticity into the model by including a new scaling factor $E(i,t)$ when computing the extinction probability of a given site i. Here, $E(i,t)$ represents environmental conditions at site i at time t. More precisely, with regional stochasticity, the probability of extinction for site i at time $t+1$ is given by

$$1 - \exp\left(-\frac{e}{q(i)E(i,t)}\right),$$

where, as in the main text, e represents the extinction parameter and $q(i)$ the fitness of the species at site i.

The value $E(i,t)$ is computed as follows. Given the parameter w that controls the degree of spatial autocorrelation, we first compute a matrix E' with dimensions corresponding to the 2-dimensional rasterization of the landscape. Starting from single cells, we recursively aggregate four neighbouring cells into a single cell, until the entire landscape consists of a single cell. Put otherwise, we recursively divide the landscape into four rectangles, until we reach rectangles that consist only of a single site. Note that each site will only be contained in a single rectangle at each level ℓ.

For each rectangle, we define a normally distributed random variable with mean $\mu = 0$ and variance σ^2. For convenience, let $X_{\ell,i}$ denote the random variable for the rectangle in which site i belongs to at level ℓ. We define the value $E'(i,t)$ as a weighted sum of the random normal variables

$$E'(i,t) = \frac{1}{W}\sum_{\ell=1}^{d} X_{\ell,i} w^{-\ell},$$

where $W = \sqrt{\sum_{\ell=1}^{d} w^{-2\ell}}$ is a normalization factor and d is the number of recursion steps required to divide the landscape.

Finally, we truncate the resulting distribution and define $E(i,t) = \min\{1, \exp(E'(i,t))\}$. Therefore, $0 < E(i,t) \leq 1$. That is, we are only interested in environmental stochasticity which increases the extinction probablity, or put otherwise, decreases the habitat quality of a site.

Properties of the distribution. Here, we derive the first and second moment of $E'(i,t)$. As we defined $\mu = 0$, it follows that the mean of $E'(i,t)$ is

$$\begin{aligned}\mathbf{E}[E'(i,t)] &= \mathbf{E}[\frac{1}{W}\sum_{\ell=1}^{d} w^{-\ell} X_{\ell,i}] \\ &= \frac{1}{W}\sum_{\ell=1}^{d} w^{-\ell}\,\mathbf{E}[X_{\ell,i}] = 0,\end{aligned}$$

since by definition $\mathbf{E}[X_{\ell,i}] = \mu = 0$ for all ℓ and i. The variance is

$$\begin{aligned}
\mathrm{Var}[E'(i,t)] &= \mathrm{Var}\left[\frac{1}{W}\sum_{\ell=1}^{d} w^{-\ell} X_{\ell,i}\right] \\
&= \sum_{\ell=1}^{d} \mathrm{Var}\left[\frac{w^{-\ell}}{W} X_{\ell,i}\right] \\
&= \sum_{\ell=1}^{d} \left(\frac{w^{-\ell}}{W}\right)^2 \mathrm{Var}[X_{\ell,i}] \\
&= \sigma^2 \sum_{\ell=1}^{d} \left(\frac{w^{-\ell}}{W}\right)^2 \\
&= \sigma^2 \frac{1}{W^2} \sum_{\ell=1}^{d} w^{-2\ell} = \sigma^2.
\end{aligned}$$

References

Gu, W., R. Heikkilä, and I. Hanski, 2002. Estimating the consequences of habitat fragmentation on extinction risk in dynamic landscapes. Landscape ecology 17:699–710.

Rybicki, J. and I. Hanski, 2013. Species–area relationships and extinctions caused by habitat loss and fragmentation. Ecology letters 16:27–38.
