# Supplementary material for: Large-Scale Habitat Corridors for Biodiversity Conservation: A Forest Corridor in Madagascar
Source: PLoS One. 2015 Jul 22;10(7):e0132126. doi: 10.1371/journal.pone.0132126 (PMC4511669; doi:10.1371/journal.pone.0132126)
Supplement: S3 File — (DOCX) [file pone.0132126.s007.docx]

**Statistical analyses of community composition and species richness in relation to environmental variables.**

We used Poisson regression to model the effects of several environmental variables on species richness in each taxonomic group: average daily temperature and precipitation during the survey period, forest cover within 5 km radius calculated from Hansen et al. [[1](#_ENREF_1)], categorical variable for altitude (below or above 1000 m asl), and whether the site is protected or not, as well as pair-wise interactions between these variables (Table A). The same covariates were used to explore variation in average body size of the species among the sites. Analyses were performed using R 3.0.2 [[2](#_ENREF_2)] .We did not use the elevation reported by Goodman and Razafindratsita [[3](#_ENREF_3)] for two reasons. First, given the geographic coordinates provided for the localities, the reported elevations do not match other sources of elevation, e.g. © Google Earth 2014 and [[4](#_ENREF_4)]. Second, there is some uncertainty concerning the coordinates of the study sites, which generates additional inaccuracy because of the steep terrain. Instead, we used the categorical variable describing whether the study site was below or above 1000 m asl. One site, Ambatambe (C5), was removed from these analyses because of extremely high precipitation (56.8 mm/day) compared to the average of 5.2 mm/day for the other localities during the survey period. Further, as there is no record of temperature for sites C8, A1 and A4, these sites were excluded from models in which temperature had a significant effect. AIC was used to select the best model. In case of two models resulting in almost similar AIC values, we explored further both models.

We report the AIC score for each model and the summary of the best model at the end of this document. We found that, in three taxonomic groups, birds, amphibians and lemurs, the average temperature has a positive effect on species richness at sites above 1000 m but a negative effect at sites below 1000 m (Figure B in S2 Fig.). These effects were highly significant in amphibians.

These results apparently reflect the fact that sampling was conducted within intact forest within a relatively narrow range of elevations. Moreover, the published coordinates are not accurate, which leads to inaccuracy in the estimation of forest cover around the sampling sites. Sampling sites at lower elevations (around 700 m asl) may have intrinsically higher species richness, but forest in lower elevations is more prone to anthropogenic disturbance and deforestation. The reasons for the significant effect of temperature, and its interaction with elevation, remain without conclusive explanation, but in any case the effect was the same in the parks and the corridor, which underscores the similarity of the forest in the corridor and in the parks.

Concerning average body size, the only significant result was obtained for lemurs, in which body size was positively related to temperature during the survey period, especially at sites below 1000 m (Figure B in S2 Fig). The P values for mT, altC, mT:altC, and PC are 0.268, 0.012,0.008, and 0.028, respectively. The latter result apparently reflects the fact that sampling was conducted within intact forest within a relatively narrow range of elevations. Moreover, the published coordinates are not accurate, which leads to inaccuracy in the estimation of forest cover around the sampling sites. Sampling sites at lower elevations (around 700 m asl) may have intrinsically higher species richness, but forest in lower elevations is more prone to anthropogenic disturbance and deforestation.

With increasing temperature at sites below 1000 m, the number of lemur species recorded declined but the ones that were present tended to be large-bodied species. Unexpectedly, average body size of lemurs was greater in the corridor than in the parks (Figure C in S2 Fig.).

Principal component analysis for each taxon did not show any clear pattern of clustering according to the three regions, Ranomafana NP, Andringitra NP and the corridor (Figures D-H in S2 Fig.). High elevation sites, such as C7, C8, A3, and A4 (Fig. 1), tend to be more similar in species composition to each other than low elevation sites.

**Table A: Description of the sampling sites.**

| Site | Id | Forest % within 5 km radius | Elevation^1^ | Elevation^2^ | Average temperature*^1^ (^o^C) | Average precipitation*^1^ (mm per day) |
| --- | --- | --- | --- | --- | --- | --- |
| Ranomena | R1 | 74.72 | 970 | 1149 | 22.65 | 16.2 |
| Vatoharanana | R2 | 84.41 | 1025 | 1091 | 19.9 | 0 |
| Marotreho | R3 | 53.77 | 910 | 821 | 24.35 | 2 |
| Andrambovato | C1 | 73.12 | 1075 | 1058 | 16.65 | 8.1 |
| Mandriandry | C2 | 72.87 | 750 | 902 | 21.4 | 6.3 |
| Ambahaka | C3 | 80.37 | 750 | 1021 | 23.2 | 5.4 |
| Vinanitelo | C4 | 83.52 | 1100 | 1017 | 20.25 | 11 |
| Ambatambe | C5 | 94.15 | 625 | 936 | 24.35 | 56.8 |
| Ankopakopaka | C6 | 78.86 | 645 | 961 | 25 | 7.4 |
| Manambolo 1 | C7 | 66.78 | 1300 | 1371 | 17 | 0 |
| Manambolo 2 | C8 | 90.75 | 1600 | 1730 | NA | 6 |
| Andringitra 1 | A1 | 98.28 | 720 | 859 | NA | NA |
| Andringitra 2 | A2 | 98.24 | 810 | 824 | 20.1 | 0 |
| Andringitra 3 | A3 | 78.98 | 1210 | 1116 | 16.45 | 0 |
| Andringitra 4 | A4 | 81.21 | 1625 | 1879 | NA | NA |

^1^ source Goodman and Razafindratsita [3]. ^2^ source Jarvis et al. [4]. * daily average values during the census

Below, the effect of varX + varY + interaction between varX and varY is denoted by varX*varY. altCB is for sites below 1000m. PCP is for sites in the parks.

Birds

Model comparison

richness ~ rad5*PC + mT*altC (AIC = 78.7)

richness ~ rad5 + PC + mT*altC (AIC = 77.19)

richness ~ rad5 + mT*altC (AIC = 75.26)

richness ~ mT*altC (AIC = 73.73)

Summary of the best model

|  | **Estimate** | **Std. error** | **z value** | **Pr(>\|z\|)** |
| --- | --- | --- | --- | --- |
| (Intercept) | 3.72159 | 0.63484 | 5.862 | 4.57e-09 *** |
| mT | 0.01913 | 0.03492 | 0.548 | 0.5839 |
| altCB | 1.64348 | 0.99290 | 1.655 | 0.0979 . |
| mT:altCB | -0.08156 | 0.04851 | -1.681 | 0.0927 . |

Amphibians

Model comparison:

richness ~ rad5*PC + mT*altC (AIC = 72.95)

richness ~ rad5 + PC + mT*altC (AIC = 71.32)

richness ~ rad5 + mT*altC (AIC = 69.34)

richness ~ mT*altC (AIC = 67.36)

Summary of the best model

|  | **Estimate** | **Std. error** | **z value** | **Pr(>\|z\|)** |
| --- | --- | --- | --- | --- |
| (Intercept) | 1.95291 | 0.92484 | 2.112 | 0.03472 * |
| mT | 0.07478 | 0.05043 | 1.483 | 0.13810 |
| altCB | 4.51610 | 1.42844 | 3.162 | 0.00157 ** |
| mT:altCB | -0.21901 | 0.06998 | -3.130 | 0.00175 ** |

Lemurs

Model comparison:

richness ~ rad5*PC + mT*altC (AIC = 56.45)

richness ~ rad5 + PC + mT*altC (AIC = 54.78)

richness ~ PC + mT*altC (AIC = 52.8)

richness ~ mT*altC (AIC = 51.08)

Summary of the best model

|  | **Estimate** | **Std. error** | **z value** | **Pr(>\|z\|)** |
| --- | --- | --- | --- | --- |
| (Intercept) | 1.75954 | 1.58112 | 1.113 | 0.2658 |
| mT | 0.02660 | 0.08686 | 0.306 | 0.7594 |
| altCB | 5.05751 | 2.77144 | 1.825 | 0.0680 . |
| mT:altCB | -0.25286 | 0.13436 | -1.882 | 0.0598 . |

Reptiles

Model comparison:

richness ~ rad5*PC + mT*altC (AIC = 60.35)

richness ~ rad5 *PC + mT + altC (AIC = 59.11)

richness ~ rad5*PC + altC (AIC = 57.29)

Summary of the best model

|  | **Estimate** | **Std. error** | **z value** | **Pr(>\|z\|)** |
| --- | --- | --- | --- | --- |
| (Intercept) | 5.4269 | 1.1189 | 4.850 | 1.23e-06 *** |
| rad5 | -3.7796 | 1.4380 | -2.628 | 0.00858 ** |
| PCP | -6.5048 | 1.6498 | -3.943 | 8.06e-05 *** |
| altCB | -0.5996 | 0.2197 | -2.730 | 0.00634 ** |
| Rad5:PCP | 8.3181 | 2.0792 | 4.001 | 6.32e-05 *** |

Micromammals

Model comparison:

richness ~ rad5*PC + mT*altC (AIC = 65.7)

richness ~ rad5*PC + mT + altC (AIC = 63.73)

richness ~ rad5*PC + altC (AIC = 62.23)

richness ~ rad5*PC (AIC = 60.6)

richness ~ rad5 + PC (AIC = 58.94)

richness ~ rad5 (AIC = 57.54)

richness ~ 1 (AIC = 56.34)

Summary of the best model

|  | **Estimate** | **Std. error** | **z value** | **Pr(>\|z\|)** |
| --- | --- | --- | --- | --- |
| (Intercept) | 2.66470 | 0.07956 | 33.49 | <2e-16 *** |

**References:**

1. Hansen MC, Potapov PV, Moore R, Hancher M, Turubanova SA, et al. (2013) High-Resolution Global Maps of 21st-Century Forest Cover Change. Science 342: 850-853.

2. R Core Team (2013) R: A language and environment for statistical computing. R Foundation for Statistical Computing, Vienna, Austria. URL <http://www.R-project.org/>.

3. Goodman SM, Razafindratsita VR, editors (2001) Inventaire biologique du Parc National de Ranomafana et du couloir forestier qui la relie au Parc National d'Andringitra: CIDST. 243 p.

4. Jarvis A, Reuter H, Nelson A, Guevara E (2006) Hole-filled seamless SRTM data V3. International Centre for Tropical Agriculture, Cali, Columbia.
