# Supplementary material for: Large-Scale Habitat Corridors for Biodiversity Conservation: A Forest Corridor in Madagascar
Source: PLoS One. 2015 Jul 22;10(7):e0132126. doi: 10.1371/journal.pone.0132126 (PMC4511669; doi:10.1371/journal.pone.0132126)
Supplement: S4 File — (DOCX) [file pone.0132126.s008.docx]

**Supporting text for running the simulations**

**Source code**

The simulations are based on two implementations of the stochastic patch occupancy model. The source code for the simulator and scripts for running the simulator using the three different modes of dispersal are located in the directories passive and active.

The first implementation implements passive dispersal (Rybicki and Hanski, 2013) and is contained in the subdirectory passive. The subdirectory active contains a new version of the simulator implementing active dispersal both with and without gap-avoidance.

**Requirements and compiling the source code**

In order to compile and run the simulation software, you need a C compiler (such as GCC) and Python 2.6. The simulation software depends on the following external libraries:

- numpy http://www.numpy.org/
- SWIG 2.0 http://www.swig.org/
- FFTW 3.3 http://www.fftw.org/
- GNU Scientific Library http://www.gnu.org/software/gsl/
- dSFMT random number generator (included) http://www.math.sci.hiroshima-u.ac.jp/~m-mat/MT/SFMT/

If you are running OS X with homebrew and pip installed, you can install the dependencies by running:

$ brew install swig fftw gsl && pip install numpy matplotlib

The software should compile on GNU/Linux and OS X operating systems.

**Overview of data files**

There are three communities of species: degraded forest specialists, forest generalists, and intact forest specialists. These are denoted by the short-hands ‘D’, ‘G’, and ‘I’, respectively. The com_*.csv files describe the parameters for the species in the respective communities. The first column represents the optimal habitat type which is either 0.25, 0.5, or 0.75 in our simulations. The second, third, fourth column denote colonization rate *c*, extinction rate *e*, and dispersal parameter *a*, respectively. For communities that use dispersal with gap-avoidance, there is a fifth column giving the number of iterative dispersal steps. In this case, the fourth column denotes the dispersal parameter for a single iteration.

The scripts for running the simulations are given in the *.sh files such as esim_XX_1.sh and tsim_X_iA_1.sh. The scripts with prefix ‘esim’ run the simulations for equilibrium analysis, whereas scripts with prefix ‘tsim’ run the transient analysis simulations. For both cases, we have included the commands for running the simulations using the forest cover data in year 2000. The label X corresponds to three different mode of dispersal: p for passive, a for active without gap-avoidance, and i for active with gap-avoidance.

In the included transient time simulations, the initial occupancy is set to be Andringitra National Park. In addition to different dispersal modes, there are cases for three species communities (D, G, and I) each with and without regional stochasticity with variance 1 and scale parameter w = 1. Furthermore, three replicates are run for each case.

The 'data' folders contain the forest cover and presence/absence data used to initialize the simulations. The files forest_cover_20XX_13.csv give the forest cover matrix for years XX = 00, 12, 38, 64, 89. The cell types are as follows: 0.25 for degraded forest, 0.75 for intact forest, and -1 for non-forest.

The initial occupancies for the species are given in the files initial_anp_13.csv and initial_rnp_13.csv. Using the first file limits the initial occupancy of species to Andringitra National Park and the second file sets the initial occupancy to Ranomafana National Park.

Finally, the files sten00.csv and sten12.csv specify the different regions of the landscape by giving a region type for each cell in the landscape. The label 0 is used for the corridor and the labels 1, 2, and 3 are for Andringitra NP and labels 4, 5, and 6 are for Ranomafana NP. The labels (in order) denote non-forest, degraded forest, and intact forest, respectively. They are used to compute the total number of occupied cell for each forest type in each park. As some forest has been lost inside the parks between the years 2000 and 2012, the two stencils are slightly different for these years. For years 2038, 2064 and 2089 we assumed that there are no changes within the parks and therefore we used sten12.csv.

**Overview of simulator scripts**

In this section, we overview the basic structure of the scripts for running the simulations. Example commands are provided in the subdirectories active and passive in the *.sh files.

The basic structure of the command line script used to execute the simulations equilibrium analysis without stochasticity is

$ python simulator.py data/forest_cover_20**X1**_13.csv com_**X2**.csv --steps **X3** -s data/sten**X4**.csv -od data/ -p **X5**_e_com**X2**_20**X1**_s**X3**_**X6**_

The bold parts denote the following values:

**X1**: 00, 12, 37, 64, and 89.

**X2**: D, G, and I for degraded forest specialist, forest generalist, and intact forest specialist, respectively.

**X3**: number of simulation time steps.

**X4**: the region stencil. We use 00 for forest_cover_2000_13.csv, and 12 otherwise.

**X5**: p, a, or i for passive dispersal, active dispersal without gap-avoidance and active dispersal with gap-avoidance, respectively.

**X6**: identifier for the replicate run.

Similarly, to execute a simulation related to the transient analysis without stochasticity we have

$ python simulator.py data/forest_cover_20**X1**_13.csv com_**X2**.csv --steps **X3** -s data/sten**X4**.csv -l data/initial_**X7**_13.csv data/initial_**X7**_13.csv data/initial_**X7**_13.csv data/initial_**X7**_13.csv data/initial_**X7**_13.csv data/initial_**X7**_13.csv data/initial_**X7**_13.csv data/initial_**X7**_13.csv -od data/ -p **X5**_t_com**X2**_**X1**_s**X3**_**X8**_**X6**_

**X7**: anp or rnp. Sets the initial condition to Andringitra or Ranomafana, respectively.

**X8**: iA or iR. Writes in the output filename whether initial condition is Andringitra or Ranomafana.

As each species use the same initial occupancy, the filename ‘data/initial_X7_13.csv' is repeated to match the number of species in 'com_X2.csv' in the stencil listing. Here we assumed there are 8 species.

To include regional stochasticity to the simulations, one can append the following parameters to the commands above:

--stochasticity 0.0 **X10** **X9**

**X9**: scale parameter w for regional stochasticity.

**X10**: variance of the normal distribution.

Finally, some further information on the different command line parameters are given by running

$ python simulator.py --help

in the spom subdirectory. For example, it is possibly to specify the number of parallel threads, seed for the random number generators, and levels of (debug and progress) output using the command line parameters for simulator.py.
